# MicroRNA-155-5p promotes neuroinflammation and central sensitization via inhibiting SIRT1 in a nitroglycerin-induced chronic migraine mouse model

**DOI:** 10.1186/s12974-021-02342-5

**Published:** 2021-12-10

**Authors:** Qianwen Wen, Yunfeng Wang, Qi Pan, Ruimin Tian, Dunke Zhang, Guangcheng Qin, Jiying Zhou, Lixue Chen

**Affiliations:** 1 Laboratory Research Center, The First Affiliated Hospital of Chongqing Medical University, 1st You Yi Road, Yu Zhong, Chongqing, 400016 China; 2 Department of Neurology, The First Affiliated Hospital of Chongqing Medical University, Chongqing, China; 3 Department of Neurology, Nanchong Central Hospital, Nanchong, China

**Keywords:** MiR-155-5p, SIRT1, Inflammation, Central sensitization, Chronic migraine, Microglia

## Abstract

**Background:**

Previous studies have confirmed that the microglial activation and subsequent inflammatory responses in the trigeminal nucleus caudalis (TNC) are involved in the central sensitization of chronic migraine (CM). MicroRNA-155-5p has been shown to modulate the polarization of microglia and participate in inflammatory processes in a variety of neurological diseases. However, its role in CM remains unclear. The purpose of this study was to determine the precise role of miR-155-5p in CM.

**Methods:**

A model of CM in C57BL/6 mice was established by recurrent intraperitoneal injection of nitroglycerin (NTG). Mechanical and thermal hyperalgesia were evaluated by Von Frey filaments and radiant heat. The expression of miR-155-5p was examined by qRT-PCR, and the mRNA and protein levels of silent information regulator 1(SIRT1) were measured by qRT-PCR, Western blotting (WB) and immunofluorescence (IF) analysis. The miR-155-5p antagomir, miR-155-5p agomir, SRT1720 (a SIRT1 activator) and EX527 (a SIRT1 inhibitor) were administered to confirm the effects of miR-155-5p and SIRT1 on neuroinflammation and the central sensitization of CM. ELISA, WB and IF assays were applied to evaluate the expression of TNF-α, myeloperoxidase (MPO), IL-10, p-ERK, p-CREB, calcitonin gene-related peptide (CGRP), c-Fos and microglial activation. The cellular localization of SIRT1 was illustrated by IF.

**Results:**

After the NTG-induced mouse model of CM was established, the expression of miR-155-5p was increased. The level of SIRT1 was decreased, and partly colocalized with Iba1 in the TNC. The miR-155-5p antagomir and SRT1720 downregulated the expression of p-ERK, p-CREB, CGRP, and c-Fos, alleviating microglial activation and decreasing inflammatory substances (TNF-α, MPO). The administration of miR-155-5p agomir or EX527 exacerbated neuroinflammation and central sensitization. Importantly, the miR-155-5p agomir elevated CGRP and c-Fos expression and microglial activation, which could subsequently be alleviated by SRT1720.

**Conclusions:**

These data demonstrate that upregulated miR-155-5p in the TNC participates in the central sensitization of CM. Inhibiting miR-155-5p alleviates neuroinflammation by activating SIRT1 in the TNC of CM mice.

## Introduction

Chronic migraine (CM) is a common debilitating neurological disorder characterized by unilateral or bilateral pulsatile headache, with at least 15 attack days per month [1, 2]. It has been listed as one of the four most serious chronic dysfunctional diseases by the WHO [3], provoking serious harm to the physical and mental health of patients [4]. Currently, central sensitization is widely accepted as one of the main pathological mechanisms of CM, which mainly manifests as the continuous increase in the excitability of neurons in the trigeminal nucleus caudalis (TNC) caused by pain stimulation [5].

Several studies have confirmed the presence of "microgliopathic pain", which might be stimulated by abnormal discharge of neurons induced by activated microglia [6–8]. Under normal physiological conditions, microglia exhibit a ‘‘resting’’ or nonactivated phenotype, acting as scavengers to maintain homeostasis [9]. However, following the recognition of infection, injury or protein aggregates, microglia become rapidly ‘‘activated’’ and transform to an ameboid morphology, releasing TNF-α, IL-1β and other inflammatory mediators [7]. These mediators then bind to specific receptors of neurons and promote central sensitization, resulting in spontaneous pain, hyperalgesia and abnormal pain [10]. Our previous research also showed that activated microglia promote central sensitization by releasing proinflammatory factors and neurotrophic factors in the TNC of CM [11–14].

In recent years, miRNAs have emerged as significant epigenetic regulators of gene expression, promoting targeted mRNA degradation or preventing protein translation [15, 16]. A large body of evidence suggests that changes in miRNA expression in the dorsal root ganglia (DRG) and spinal cord in a neuropathic pain model are related to hyperalgesia [17]. A few studies have confirmed that the downregulation of miR-155 reduces pain thresholds and proinflammatory cytokines in several pain models [18, 19]. A clinical study showed multi-fold higher miR-155 levels in circulating blood in migraine patients than in healthy controls [20], suggesting that miR-155 might participate in the pathological mechanism of migraine. MiR-155 has been demonstrated to play a proinflammatory role during microglial activation by inhibiting anti-inflammatory proteins (SIRT1, SOCS1, SHIP1, etc.) [21]. SIRT1 has been accepted as a target gene of miR-155 that alleviates major depressive disorder [22]. SIRT1 is an NAD^+^-dependent histone deacetylase that regulates inflammation, metabolism and other physiological processes, and several studies have shown its positive effects against neuropathic pain [9, 23]. Therefore, considering that the potential mechanism of miR-155 in migraine is still unclear and the possible connection between miR-155 and inflammation, we hypothesized that miR-155-5p might induce the microglial activation and inflammatory responses by downregulating the expression of SIRT1, resulting in central sensitization and hyperalgesia in a mouse model of chronic migraine.

In this study, we innovatively proposed that miR-155-5p might be involved in the pathological mechanism of chronic migraine. The upregulation of miR-155-5p and its effects on hyperalgesia were confirmed in our model. Our data revealed that miR-155-5p regulates the central sensitization of chronic migraine by activating neuroinflammation via downregulating SIRT1.

## Materials and methods

### Animals

**Fig. 1:**
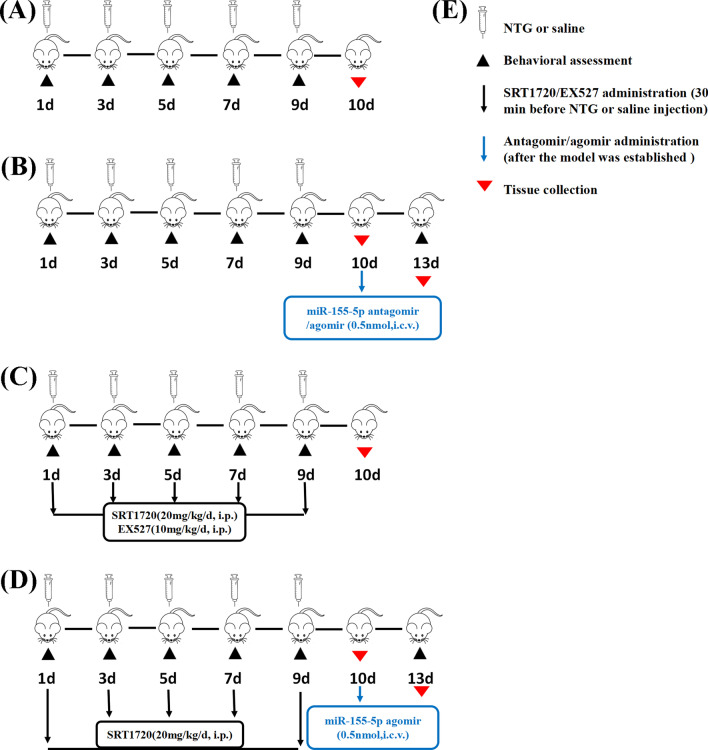
Time schedule of drug administration, behavioral testing, and sample collection. **A** The Sham and NTG group. **B** The NTG + miR-155-5p antagomir and NTG + agomir group. **C** The NTG + SRT1720 and NTG + EX527 group. **D** The NTG + miR-155-5p agomir + SRT1720 group. **E** The label of the symbols. Behavioral assessments were conducted before NTG injection or 3 days after antagomir/agomir administration. Tissues were collected within 24 h after the last NTG injection or 3 days after miR-155-5p antagomir/agomir administration

Male C57BL/6 mice (weighing 18–20 g, aged 8–10 weeks) were acquired from the Experiment Animal Center of Chongqing Medical University (Chongqing, China). The mice were housed with an alternating light–dark cycle of 12 h at the standard conditions of 22 ± 2 °C and a relative humidity of 50 ± 10% and were provided with sufficient food and water. All animal experimental procedures were approved by the Animal Care and Use Committee at Chongqing Medical University and were conducted in accordance with the National Institutes of Health Guide for the Care and Use of Laboratory Animals. We tried our best to reduce the number of animals used in the experiment and minimize animal suffering. Before the experiment, all animals were allowed to acclimatize to the environment for one week and were then randomly assigned to different experimental groups according to the random number sequence generated by the Excel software. During the treatment, the animals were weighed every day, and no adverse effects were observed. All detailed time courses of the experiment (drug administration, behavioral testing, and sample collection) are described in Fig. 1.

### Establishment of the chronic migraine model

A chronic migraine model was established as previously described [24]. NTG (Runhong, Henan, China) was prepared from a stock solution of 5.0 mg/ml dissolved in alcohol, citric acid, sodium citrate and water. Before administration, NTG was freshly diluted to 1.0 mg/ml with 0.9% saline. Mice received repeated intraperitoneal injections of diluted nitroglycerin or an equal volume of vehicle every other day for 9 days (five times in total), at a dose of 10 mg/kg.

### Drug administration

According to the experimental requirements, mice were randomly divided into the following groups: (1) Sham group, (2) NTG group, (3) NTG + miR-155-5p anta NC group, (4) NTG + miR-155-5p antagomir group, (5) NTG + miR-155-5p ago NC group, (6) NTG + miR-155-5p agomir group, (7) NTG + vehicle group, (8) NTG + SRT1720 group, (9) NTG + EX527 group, and (10) NTG + miR-155-5p agomir + SRT1720 group. In groups (3)–(6), the miR-155-5p antagomir, agomir or their scrambled sequence control (GenePharma, Shanghai, China) were separately administered after establishing the NTG-induced mouse model. Mice were placed under deep anesthesia and positioned in a stereotaxic apparatus (ST-51603; Stoelting Co, Chicago, IL, USA), and 1 μL of phosphate-buffered saline (PBS) containing 0.5 nmol miR-155-5p antagomir or agomir was injected over 5 min into the left lateral cerebral ventricle (1.6 mm lateral and 1 mm anteroposterior to the bregma) [25, 26]. The negative control was a scrambled sequence of the antagomir or agomir. SRT1720 (MedChemExpress/MCE, USA) is a selective SIRT1 activator that is 230-fold less potent for SIRT2 and SIRT3. EX527 (MedChemExpress/MCE, USA) is an effective and selective SIRT1 inhibitor. In group (8), SRT1720 was intraperitoneally administered before NTG injection at a dose of 20 mg/kg every other day for 9 days (five times in total), in an identical manner to NTG treatment. In group (9), EX527 was intraperitoneally administered at a dose of 10 mg/kg. The doses of these two drugs were determined according to the literature [27–30].

### Sensory sensitivity testing

Central sensitization manifests as cutaneous allodynia involving the craniofacial and noncraniofacial regions. Therefore, periorbital and hindpaw hyperalgesia were measured to examine the characteristics of the CM mouse model. All behavioral assessments were conducted before NTG injection in a quiet environment with low light and low noise conditions between 09:00 and 15:00. Before the experiment, the mice received 3 days of training. Each experiment was performed by the same investigators, who were blinded to the groups.

#### Paw withdrawal latency

The plantar test apparatus (Techman PL-200, Chengdu, China) was used to measure the thermal thresholds as previously described [31]. After 30 min of acclimatization in a smooth, glass-floored transparent cage, the center of each mouse’s hindpaw was stimulated with infrared radiation. Once the hindpaw was moved, the stimulus stopped, and the withdrawal latency was automatically recorded. The radiant heat intensity was calibrated to produce a basic withdrawal latency of 10–12 s in normal mice. To prevent tissue damage, an automatic 20-s cut-off was set [32]. Each mouse was examined three times, and the mean latencies with an interval of at least 5 min were calculated.

#### Paw mechanical threshold

Paw mechanical threshold was measured as previously described [33, 34]. Mice were separately placed on a wire mesh platform for 30 min and allowed to habituate. A range of eight Von Frey monofilaments (0.04–2 g) was applied to the center of the plantar surface of the hindpaw, with an initial stimulation strength of 0.4 g. A positive response was defined as shaking, withdrawal, or licking of the paw. Each filament was applied to the skin for 3 s, with an interval of at least 1 min. Testing of the mechanical threshold and calculations were performed using the up-and-down method, which was first described by Chaplan et al. [35]. The hindpaw was stimulated five times, and a positive response to three or more of the five stimulations was considered a positive response for that filament. The filament strength was increased if there was no response to this stimulation; otherwise, the filament strength was decreased.

#### Periorbital mechanical threshold

For periorbital measures, mice were tested in 4 oz paper cups that were placed in the plexiglass box. Then, the monofilament was applied to the periorbital region caudal to the eyes and near the midline. The up- and-down method was used to determine the periorbital withdrawal threshold to mechanical stimulation as previously described for the paw mechanical threshold. Scratching the face with front claws, retracting quickly or vocalizing due to monofilament stimulation were considered to be positive reactions.

### Quantitative real-time reverse transcriptase polymerase chain reaction (qRT-PCR)

After the mice were euthanized with 10% chloral hydrate, TNC tissue was isolated and immediately stored in liquid nitrogen for qRT-PCR. Total RNA was extracted by RNAiso Plus reagent (Takara, Tokyo, Japan). The RNA concentration was spectrophotometrically quantified using a NanoDrop spectrophotometer (Thermo, Waltham, USA). Next, reverse transcription of miR-155-5p was conducted by a Mir-X™ miRNA First-Strand Synthesis Kit (Takara, Tokyo, Japan), and reverse transcription of SIRT1, CD86, iNOS, CD206 and Arg1 was performed by a PrimeScript™ RT Reagent Kit (Takara, Tokyo, Japan). mRNA expression was analyzed on a CFX96 Touch thermocycler (Bio-Rad, USA) using SYBR Premix Ex Taq TM II (Takara, Tokyo, Japan). The amount of mRNA was normalized to GAPDH or U6 using the 2^−ΔΔCT^ method. Primer sequences are listed in Table 1.

**Table 1:** Primers used in real-time PCR reactions

Gene	Forward primer (5′ → 3′)	Reverse primer (5′ → 3′)
miR-155-5p	GCTTCGGTTAATGCTAATCGTG	CAGAGCAGGGTCCGAGGTA
U6	TGCGGGTGCTCGCTTCGGCAGC	CCAGTGCAGGGTCCGAGGT
SIRT1	TGTTTCCTGTGGGATACCTGA	TGAAGAATGGTCTTGGGTCTTT
GAPDH	ACGGCAAGTTCAACGGCACAG	GACGCCAGTAGACTCCACGACA
CD86	TCTGCCGTGCCCATTTACAAAGG	TGTGCCCAAATAGTGCTCGTACAG
iNOS	ACTCAGCCAAGCCCTCACCTAC	TCCAATCTCTGCCTATCCGTCTCG
CD206	TGATTGGTGGCAATTCACGAGAGG	AACAGGCAGGGAAGGGTCAGTC
Arg1	GGCAACCTGTGTCCTTTCTCCTG	GGTCTACGTCTCGCAAGCCAATG

### Western blot analysis

Fresh TNC tissue was homogenized by a grinding machine (Jingxin, Shanghai, China) with radioimmunoprecipitation (RIPA) lysis buffer (Beyotime, Shanghai, China) containing phenylmethylsulphonyl fluoride (PMSF, Beyotime, Shanghai, China). The machine was precooled to – 30 ℃, and tissues were homogenized for 10 s each time (three times in total with an interval of 10 s). A bicinchoninic acid (BCA) protein analysis kit (Beyotime, Shanghai, China) was used to quantify the protein concentration. Equal amounts of protein (25 μg per lane) were separated on a 10% or 12% SDS–PAGE gel (Beyotime, Shanghai, China) and electrotransferred to a polyvinylidene difluoride (PVDF) membrane (Millipore, USA). The membranes were blocked for 2 h at room temperature with 5% skim milk in Tris-buffered saline containing 0.1% Tween 20 and incubated overnight at 4 °C with the primary antibodies listed in Table 2. The next day, the membranes were washed with TBST three times for 10 min and then incubated with the corresponding peroxidase-conjugated secondary antibodies for 1 h at room temperature. GAPDH and β-actin were used as endogenous controls to normalize the target proteins. The immunoblotted proteins were visualized by an imaging system (Fusion, Germany) using the ECL Plus Chemiluminescence kit (ZEN-BIOSCIENCE, Chengdu, China). The antibodies used are listed in Table 2.

**Table 2:** Antibodies used in Western blotting and immunofluorescence analysis

Antibody	Manufacturer	Catalog number	Host	Dilution
*For Western blotting analysis*				
SIRT1	Abcam, UK	ab110304	Mouse	1:3000
CGRP	Abcam, UK	ab189786	Rabbit	1:3000
c-Fos	Abcam, UK	ab134122	Rabbit	1:1000
p-ERK	CST, USA	4370 T	Rabbit	1:1000
ERK	CST, USA	4695 T	Rabbit	1:2000
p-CREB-S133	Abcam, UK	ab32096	Rabbit	1:3000
CREB	CST, USA	9197 T	Rabbit	1:1000
Iba1	Abclonal, China	A1527	Rabbit	1:1000
iNOS	Proteintech, USA	18985-1-AP	Rabbit	1:1000
GAPDH	ZEN-BIOSCIENCE, China	200306-7E4	Mouse	1:5000
β-actin	ZSGB-Bio, China	TA-09	Mouse	1:3000
Anti-rabbit IgG (HRP)	ZEN-BIOSCIENCE, China	511203	Goat	1:5000
Anti-mouse IgG (HRP)	ZEN-BIOSCIENCE, China	511103	Goat	1:5000
*For immunofluorescence staining*				
SIRT1	Abcam, UK	ab110304	Mouse	1:50
Iba1	Wako, Japan	019-19741	Rabbit	1:400
NeuN	CST, USA	24307 T	Rabbit	1:400
GFAP	Wanleibio, China	WL0836	Rabbit	1:50
CGRP	Santa Cruz, USA	sc-57053	Mouse	1:100
c-Fos	Novus Biologicals, USA	NBP2-50057SS	Rabbit	1:5000
Alexa Fluor 488 goat anti-mouse IgG	Beyotime, China	A0428	Goat	1:500
Cy3-conjugated goat anti-rabbit IgG	Beyotime, China	A0516	Goat	1:500

### Immunofluorescence staining

After being anesthetized with 10% chloral hydrate, the mice were transcardially perfused with 60 ml of PBS (pH 7.2–7.4) and 60 ml of 4% paraformaldehyde (PFA). PFA (4%) was used to postfix the isolated tissues for 12 h at 4 °C, and then 20% and 30% sucrose solutions were used to dehydrate. Sections (12 μm) were collected after the tissues were sliced coronally by a freezing microtome (Leica, Japan). The sections were subjected to antigen retrieval by being boiled in sodium citrate buffer (Beyotime, Shanghai, China) and permeabilized with 0.3% Triton X-100 (Beyotime, Shanghai, China) at 37 °C for 10 min. Then, 10% goat serum (Boster, Wuhan, China) was used to block the slices at 37 °C for 30 min. The sections were incubated overnight at 4 °C with primary antibodies diluted in 1% PBS. The next day, the sections were incubated with secondary antibodies for 60 min and 4′,6-diamidino-2-phenylindole (DAPI) for 10 min at 37 °C. After being sealed with 50% glycerol, the sections were visualized by a confocal laser scanning fluorescence microscope (ZEISS, Germany). The negative control section was treated with PBS instead of the primary antibody, and no positive signal was seen. The antibodies used are listed in Table 2.

### Immunofluorescence imaging data analysis

The TNC region was determined based on morphology under a light microscope according to the Mouse Brain Atlas [36]. The immunofluorescence images were analyzed by ImageJ software (version 1.8.0_112). Four to six sections per mouse (6 mice total) were randomly selected from each group. Mean optical density (OD) values were measured to represent the levels of CGRP and SIRT1 at a magnification of × 50 or × 200, respectively, and the background intensity was calculated by correcting the intensity of the stained area. The squared images (320 × 320 µm^2^) in the superficial layer of the TNC were taken at 200 × magnification to quantify the number of c-Fos- and Iba1-positive cells. The total and average length of microglial processes directly extended from the cell body were analyzed by Neuron J (an ImageJ plug-in). Image collection and analysis were performed by an experimenter who was blinded to the experimental groups.

### Enzyme-linked immunosorbent assay (ELISA)

The levels of TNF-α, MPO, and IL-10 in the TNC were measured by specific ELISA kits (Meike, Shanghai, China). The tissue was weighed, and PBS (pH 7.2–7.4, 0.01 mol/L) was added at a 1:9 proportion and homogenized at -30 °C. Then, the slurry was centrifuged at 5000 rpm for 15 min, and the supernatant was collected. According to the instructions, samples were added to the corresponding microenzyme plate wells and bound with specific antibodies. Then, HRP-conjugated reagent was added, the plate was sealed with a membrane and incubated for 60 min at 37 °C. Next, chromogen solutions A and B were added to each well after the wells were washed with washing buffer. Chromogen solutions A and B were protected from the light for 15 min at 37 ℃. Finally, stop solution was added to each well, and the optical density (OD) was measured at 450 nm.

### Statistical analysis

Data in this article are presented as the mean ± SEM. GraphPad Prism 8.0.1 (GraphPad Software Inc., San Diego, CA, USA) was applied for graphs generation and statistical evaluation. Before statistical analysis, the Kolmogorov–Smirnov test was applied to analyze the date for normality and lognormality. Independent-sample t tests were performed to assess differences between two groups. One-way analysis of variance (ANOVA) followed by the Dunnett’s multiple comparison test was used to compare multiple groups. Two-way ANOVA with the Bonferroni post hoc test was performed to analyze behavioral data. Two-tailed tests were applied in all statistical analyses. Values of *p* < 0.05 were considered to be statistically significant.

## Results

### The administration of NTG caused hyperalgesia and upregulated the levels of CGRP and c-Fos

**Fig. 2:**
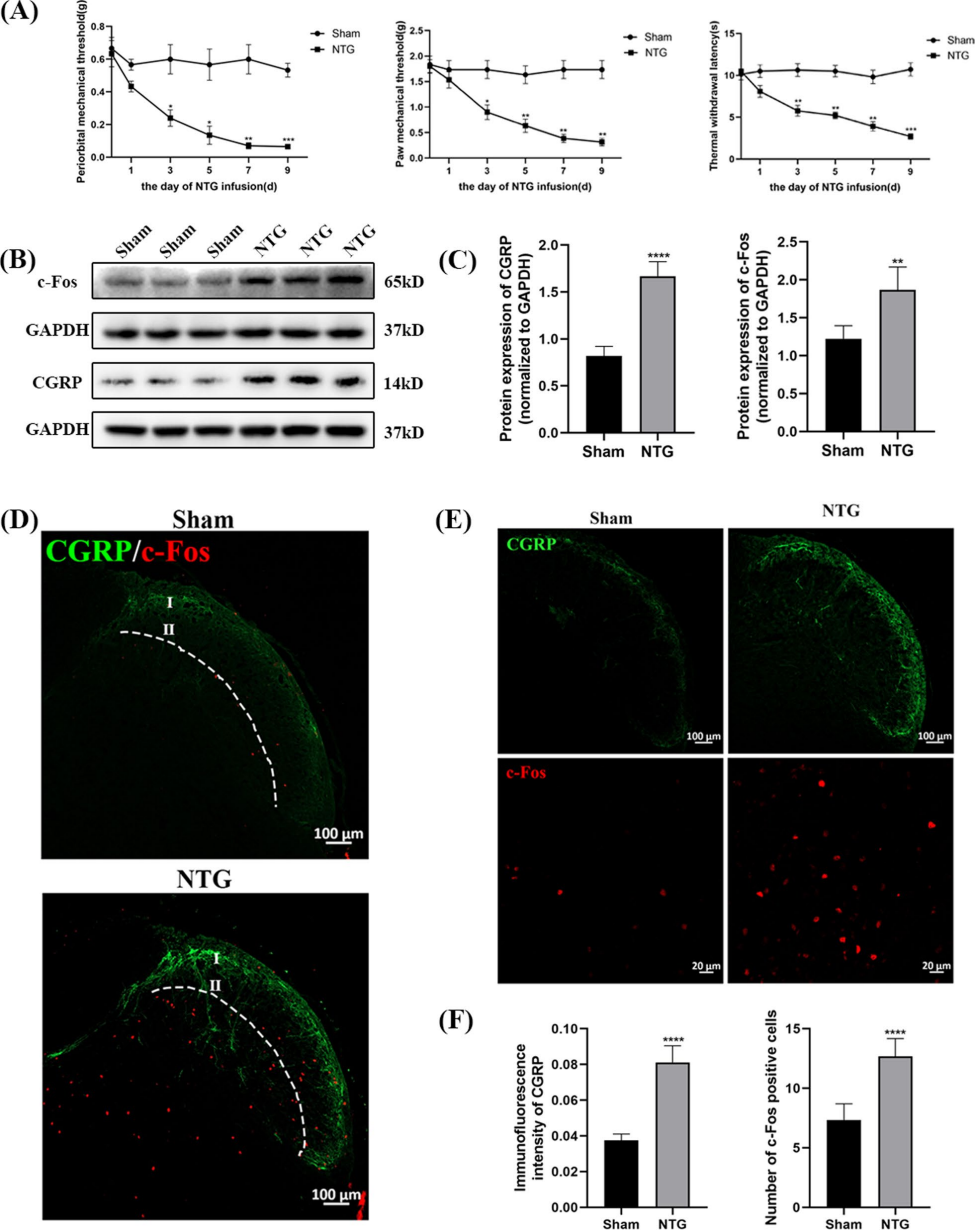
Recurrent NTG injection induced hyperalgesia and upregulated the expression of CGRP, c-Fos. **A** The periorbital and paw mechanical threshold and thermal withdrawal latency during the injection of NTG. **B**, **C** The protein expression of CGRP and c-Fos in the TNC. **D** Counting of c-Fos was restricted to laminae I and II of the TNC region. **E**, **F** Immunofluorescence staining images of CGRP and c-Fos in the TNC. (n = 6–8 in each group; scale bar = 20/100 μm; *p < 0.05, **p < 0.01, ***p < 0.001 and ****p < 0.0001 compared with the Sham group)

After the NTG-induced mouse model of CM was established (Fig. 1A), mechanical allodynia, thermal hyperalgesia and the protein levels of CGRP and c-Fos were measured to evaluate the reliability of our model. Compared with those of the Sham group, the periorbital and paw mechanical threshold and withdrawal latency were notably decreased in the NTG group (Fig. 2A). The pain thresholds decreased gradually in a time-dependent manner, and a significant decrease occurred after the second infusion. However, no significant difference was observed in the Sham group. CGRP is involved in the occurrence and maintenance of migraine [37]. c-Fos is regarded as a sign of neuronal activation in response to nociceptive stimulation, which is closely related to the pathological mechanism of migraine [38]. Western blot analysis showed increased expression of CGRP (*p* < 0.0001) and c-Fos (*p* = 0.0011) after repeated injections of NTG, and consistent results were found by immunofluorescence staining (Fig. 2B–F). Counting of c-Fos was restricted to laminae I and II of the TNC (Fig. 2D). These results indicated that we established a successful and reliable mouse model of CM, which could be used in the following experiments.

### The level of miR-155-5p was increased in the TNC after recurrent NTG administration, and the mRNA and protein levels of SIRT1 were decreased

**Fig. 3:**
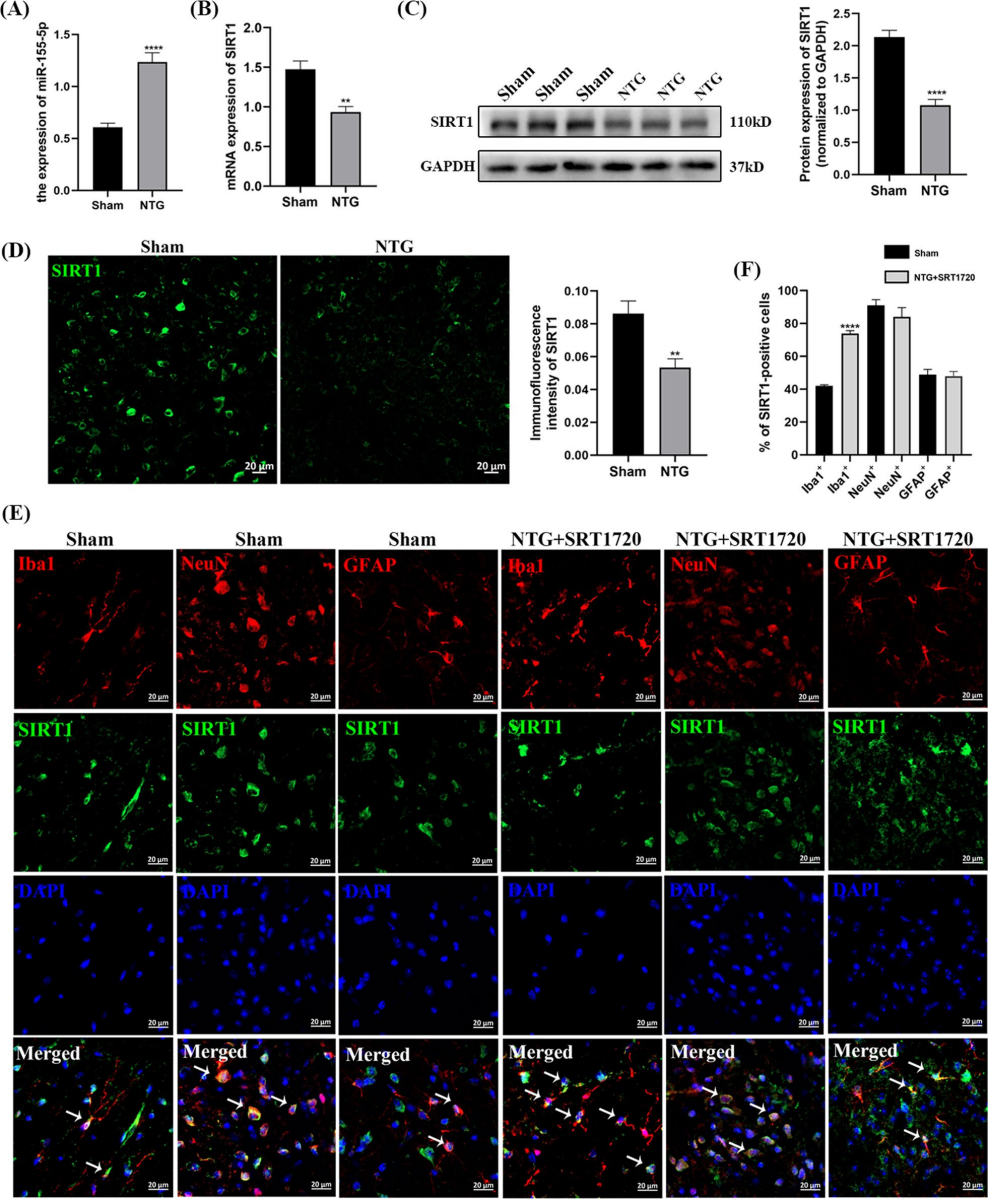
The administration of NTG increased miR-155-5p level and decreased the expression of SIRT1. **A** The expression level of miR-155-5p. **B** The mRNA expression of SIRT1. **C** The protein level of SIRT1. **D** The immunofluorescence intensity of SIRT1. **E**, **F** SIRT1 was partly coexpressed with Iba1, NeuN and GFAP in the TNC. After SRT1720 (a SIRT1 activator) treatment, the coexpression of SIRT1 and Iba1 increased, while the co-localization of SIRT1 and GFAP did not change. (n = 6–8 in each group; scale bar = 20 μm; **p < 0.01 and ****p < 0.0001 compared with the Sham group)

To explore the role of miR-155-5p and SIRT1 in chronic migraine, the expression of miR-155-5p was measured by qRT-PCR, and SIRT1 was measured by qRT-PCR, WB and IF. Compared with that in the Sham group, miR-155-5p in the NTG group increased significantly (*p* < 0.0001), and the mRNA expression of SIRT1 (*p* = 0.0012) decreased (Fig. 3A, B). To comprehensively validate changes in SIRT1 expression, WB and IF were employed to determine the protein level of SIRT1, and there were distinctly downregulated SIRT1 levels (*p* < 0.0001 and *p* = 0.0062) in the NTG group (Fig. 3C, D). In Fig. 3E and F, the localization of SIRT1 in the TNC was analyzed by double immunofluorescence staining, and the results showed that SIRT1 was partly coexpressed with Iba1 (a marker of microglia), NeuN (a marker of neuron) and GFAP (a marker of astrocytes). What’s more, after the administration of SRT1720 (a SIRT1 activator), the coexpression of SIRT1 and Iba1 increased, while the co-localization of SIRT1 and GFAP did not change. These combined data suggest that miR-155-5p and SIRT1 might be associated with the pathological mechanism of CM.

### The application of the miR-155-5p antagomir and agomir affected hyperalgesia and central sensitization in the TNC of CM mice

**Fig. 4:**
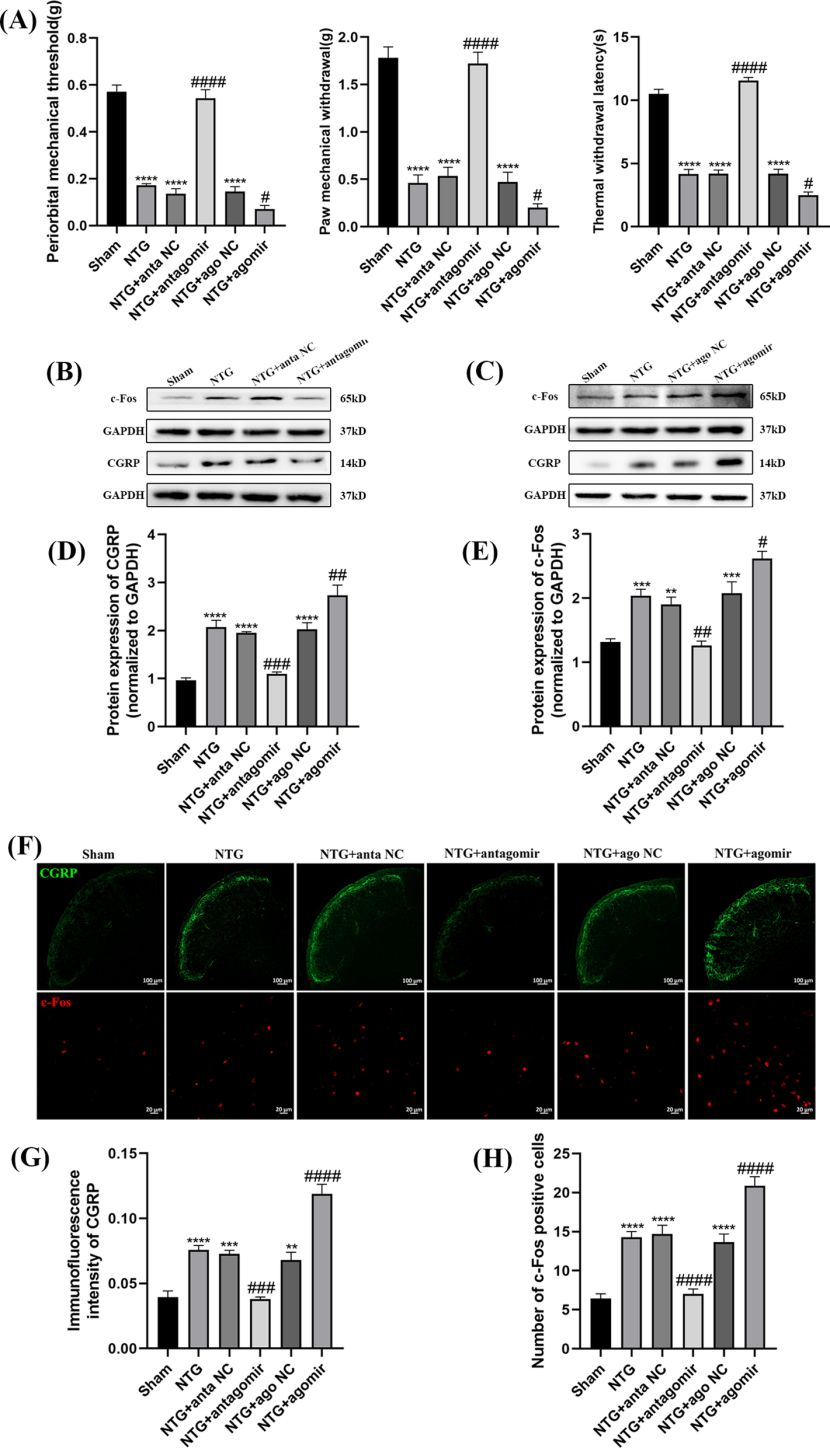
The effects of miR-155-5p antagomir and agomir on hyperalgesia and the expression of CGRP, c-Fos. **A** The mechanical and thermal pain thresholds in different groups. Compared with the Sham group, pain thresholds significantly decreased in the NTG group. Compared with the NTG + anta NC group, the pain thresholds were notably increased in the NTG + miR-155-5p antagomir group. The pain thresholds in the NTG + miR-155-5p agomir group were reduced compared to NTG + ago NC group. **B**, **C**, **D**, **E** The protein expression of CGRP and c-Fos. Compared with the Sham group, the protein levels of CGRP and c-Fos notably increased in NTG group. Compared with the NTG + anta NC group, the expression of CGRP and c-Fos were decreased in the NTG + miR-155-5p antagomir group. The expression of CGRP and c-Fos in the NTG + miR-155-5p agomir group was significantly increased compared to NTG + ago NC group. **F**, **G**, **H** Immunofluorescence staining images of CGRP and c-fos. Compared with the Sham group, the immunofluorescence intensity of CGRP and number of c-Fos-positive cells evidently increased in NTG group. Compared with the NTG + anta NC group, the immunofluorescence intensity of CGRP and number of c-Fos-positive cells were markedly decreased in the NTG + miR-155-5p antagomir group. The immunofluorescence intensity of CGRP and number of c-Fos-positive cells in the NTG + miR-155-5p agomir group were increased compared to NTG + ago NC group. There were no significant differences among the NTG, NTG + anta NC and NTG + ago NC groups. (n = 6–8 in each group; scale bar = 20/100 μm; **p < 0.01, ***p < 0.001 and ****p < 0.0001 compared with the Sham group; #*p* < 0.05, ##*p* < 0.01, ###*p* < 0.001 and ####*p* < 0.0001 compared with the NTG + NC groups)

To investigate the effects of miR-155-5p on hyperalgesia and central sensitization, a miR-155-5p antagomir or agomir was delivered to the lateral cerebral ventricle (Fig. 1B). The thresholds of periorbital mechanical pain, hindpaw mechanical pain, and hindpaw thermal pain in the NTG group were significantly lower than those in the Sham group (Fig. 4A). The miR-155-5p antagomir reversed the effect of NTG on mechanical and thermal allodynia, while injection of the miR-155-5p agomir exacerbated hyperalgesia (Fig. 4A). As shown in Fig. 4B–E, the increased levels of CGRP (*p* = 0.0002) and c-Fos (*p* = 0.0015) induced by NTG were abolished by the miR-155-5p antagomir, while treatment with the miR-155-5p agomir had a negative effect on attenuating central sensitization. The changes in the immunofluorescence intensity of CGRP and the number of c-Fos-positive cells were consistent with the WB results (Fig. 4F–H). There was no significant difference among the NTG, NTG + ago NC and NTG + anta NC groups. These results confirm that inhibiting miR-155-5p could attenuate hyperalgesia and central sensitization in chronic migraine.

**Fig. 5:**
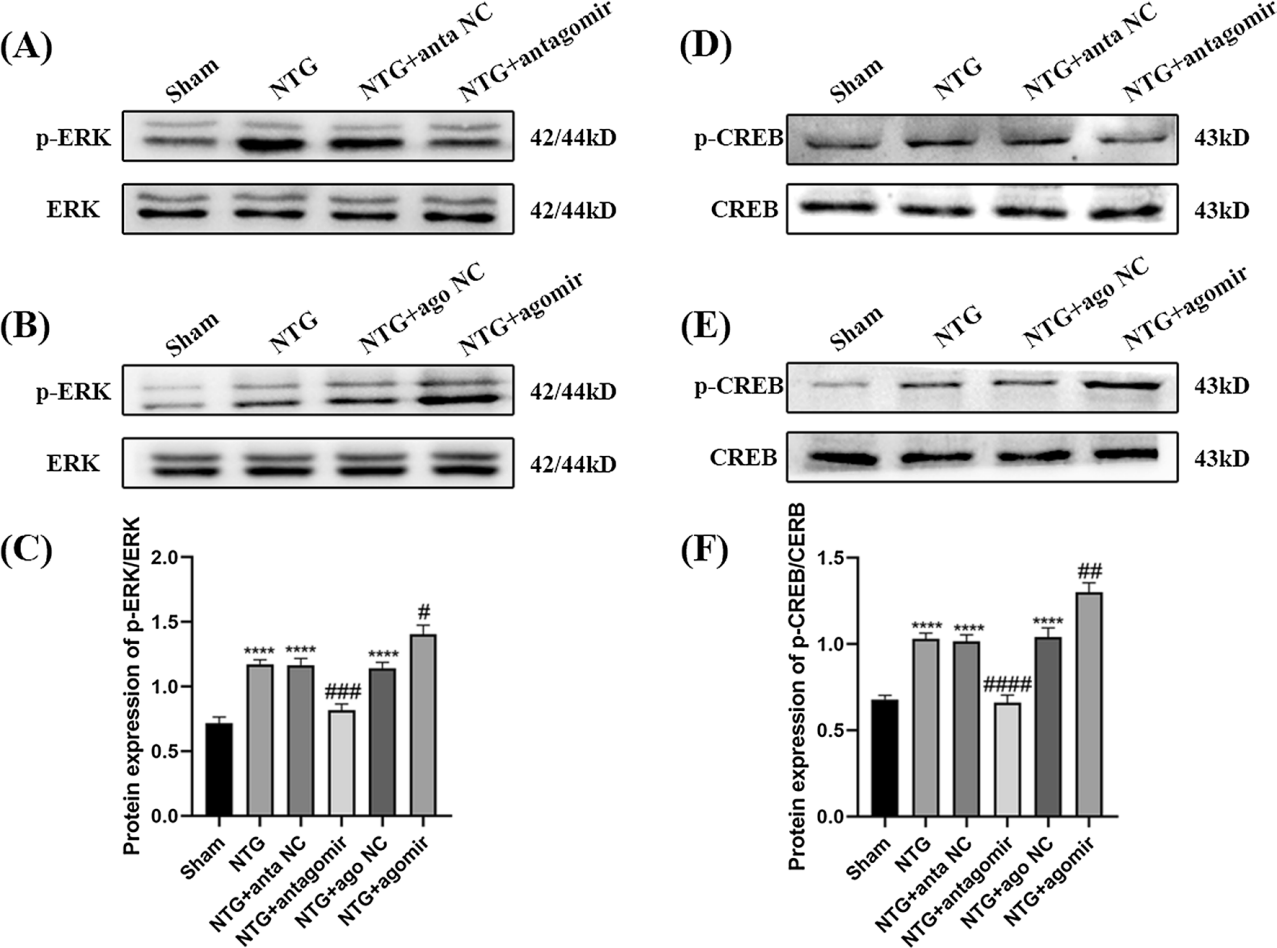
The effects of miR-155-5p antagomir and agomir on the protein levels of p-ERK and p-CREB. **A**, **B**, **C**, **D**, **E**, **F** Compared with the Sham group, the expression of p-ERK and p-CREB-S133 increased in the NTG group. Compared with the NTG + anta NC group, the expression of p-ERK and p-CREB-S133 were notably decreased in the NTG + miR-155-5p antagomir group. The expression of p-ERK and p-CREB-S133 in the NTG + miR-155-5p agomir group were significantly increased compared to NTG + ago NC group. The total amount of ERK and CREB remained stable among the Sham, NTG, NTG + anta NC, NTG + antagomir, NTG + ago NC and NTG + agomir groups. (n = 6–8 in each group; ****p < 0.0001 compared with the Sham group; ##*p* < 0.01, ###*p* < 0.001 and ####*p* < 0.0001 compared with the NTG + NC groups)

The ERK/CREB signal transduction pathway is associated with neuronal excitation and is related to the occurrence and maintenance of migraine [39, 40]. The protein levels of p-ERK (*p* < 0.0001) and p-CREB (*p* < 0.0001) were markedly upregulated after intraperitoneal injection of NTG, while no significant differences were observed in total CREB and ERK levels (Fig. 5). After treatment with the miR-155-5p antagomir, the p-ERK (*p* = 0.0002) and p-CREB (*p* < 0.0001) expression levels induced by NTG were both substantially reduced, while the protein levels of p-ERK (*p* = 0.0154) and p-CREB (*p* = 0.0018) in the NTG + miR-155-5p agomir group were higher than those in the NTG + ago NC group. The scrambled sequence did not affect the phosphorylation of ERK or CREB. Collectively, these results suggest that phosphorylated ERK and CREB might participate in miR-155-5p regulated central sensitization.

### miR-155-5p inhibited the level of SIRT1

**Fig. 6:**
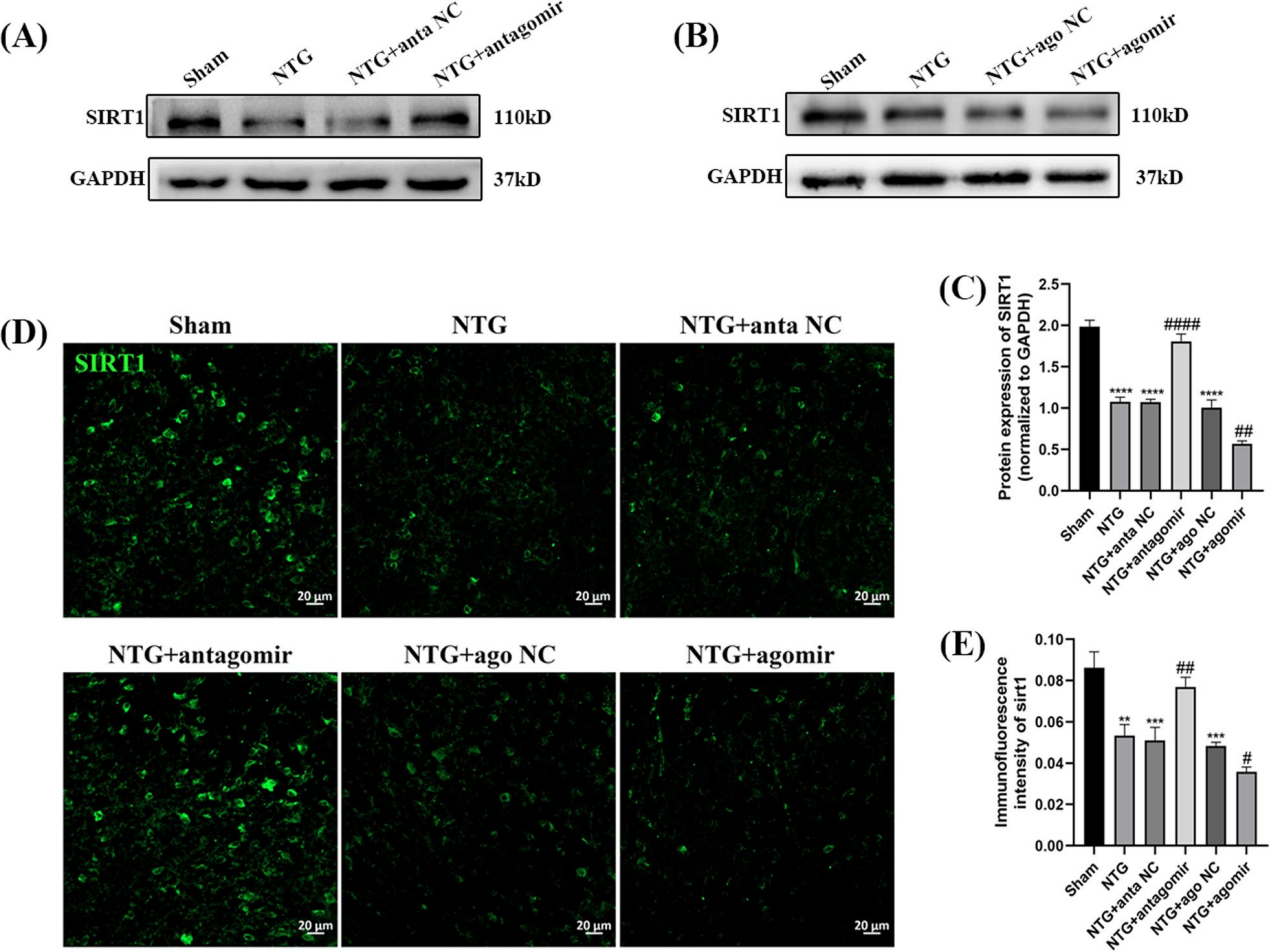
The effects of miR-155-5p antagomir and agomir on the expression of SIRT1. **A**, **B**, **C** Compared with the Sham group, the protein level of SIRT1 notably decreased in NTG group. Compared with the NTG + anta NC group, the expression of SIRT1 was increased in the NTG + miR-155-5p antagomir group. The expression of SIRT1 in the NTG + miR-155-5p agomir group was significantly decreased compared to NTG + ago NC group. **D**, **E** The immunofluorescence intensity of SIRT1 in the NTG group was lower compared to the Sham group. MiR-155-5p antagomir and agomir resulted in similar effects on SIRT1 compared with the WB results. There were no significant differences among the NTG, NTG + anta NC and NTG + ago NC groups. (n = 6–8 in each group; scale bar = 20 μm; **p < 0.01, ***p < 0.001 and ****p < 0.0001 compared with the Sham group; #*p* < 0.05, ##*p* < 0.01 and ####*p* < 0.0001 compared with the NTG + NC groups)

To explore the potential mechanism of miR-155-5p in chronic migraine, WB and immunofluorescence were used to measure the protein level of SIRT1. As shown in Fig. 6A–C, administration of the miR-155-5p antagomir abrogated the downregulation of SIRT1 (*p* < 0.0001) induced by NTG, while the expression of SIRT1 (*p* = 0.0021) in the NTG + miR-155-5p agomir group was lower than that in the NTG + ago NC group. The immunofluorescence results were consistent with the WB results (Fig. 6D, E). There was no significant difference among the NTG + anta NC, NTG + ago NC and NTG groups. These results suggest that miR-155-5p downregulates the expression level of SIRT1.

### Treatment with the miR-155-5p antagomir and agomir affected microglial activation

**Fig. 7:**
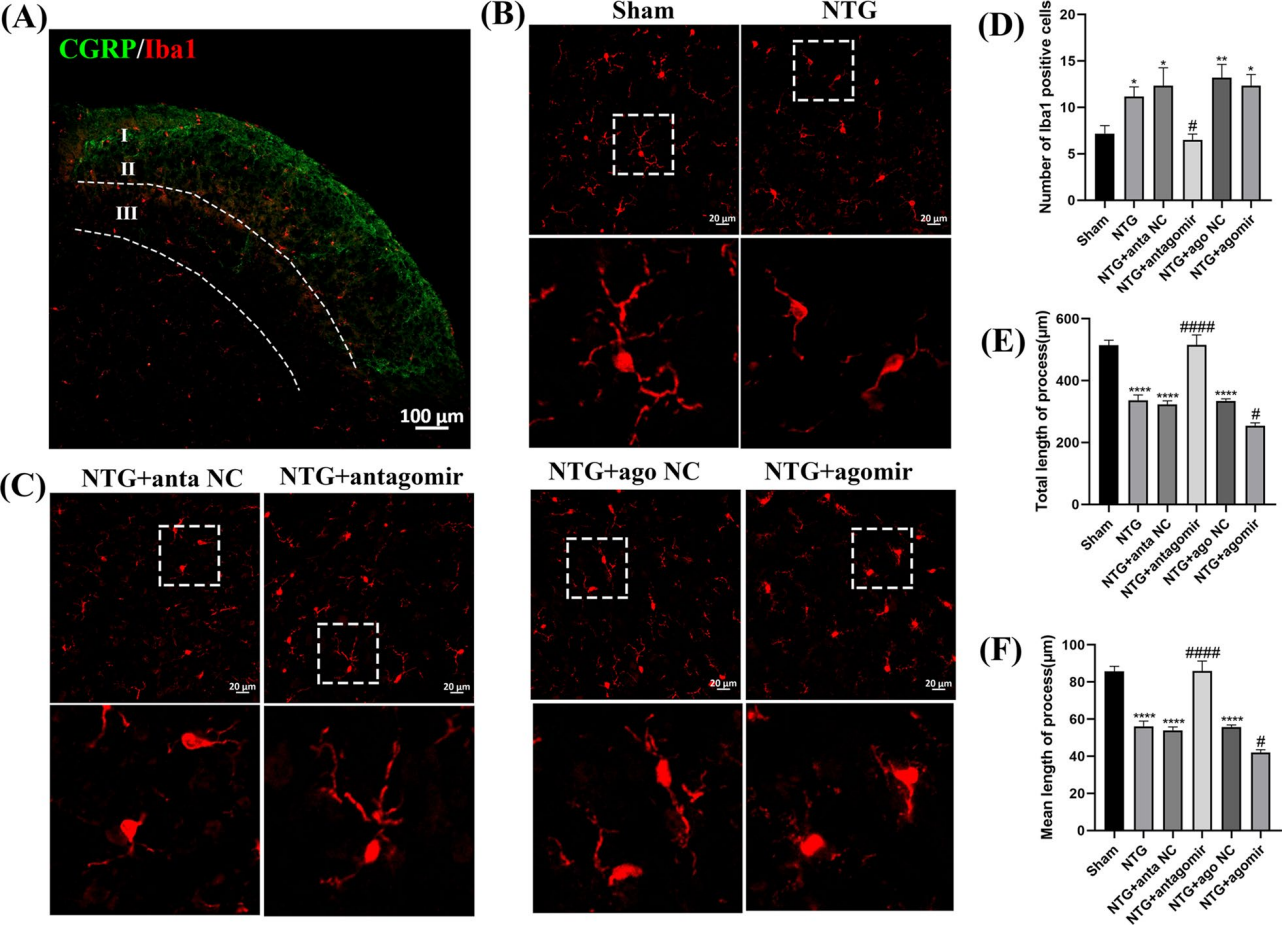
The effects of miR-155-5p antagomir and agomir on proliferation and morphological changes of microglia. **A** Immunofluorescence analysis of Iba1 was restricted to laminae I, II and III of the TNC region. The NTG group was selected to present the partition more clearly. **B**, **C**, **D**, **E**, **F** Compared with the Sham group, the number of Iba1-immunoreactive cells increased, while the total and mean length decreased in NTG group. Compared with the NTG + anta NC group, the Iba1-immunoreactive cells distinctly decreased, while the total and mean length were higher in NTG + miR-155-5p antagomir group. The total and mean length decreased in the NTG + miR-155-5p agomir group compared to NTG + ago NC group, and no significant difference was observed in Iba1-immunoreactive cells between the two groups. There were no significant differences among the NTG, NTG + anta NC and NTG + ago NC groups. (n = 6–8 in each group; *p < 0.05, **p < 0.01 and ****p < 0.0001 compared with the Sham group; #*p* < 0.05 and ####*p* < 0.0001 compared with the NTG + NC groups)

To determine whether miR-155-5p could activate microglia in CM, immunofluorescence staining was applied to observe the proliferation and morphological changes in microglia. Immunofluorescence analysis of Iba1 was restricted to laminae I, II and III of the TNC region (Fig. 7A). Repeated administration of NTG markedly increased the number of Iba1-immunoreactive cells (*p* = 0.0149) and decreased the total (*p* < 0.0001) and mean length (*p* < 0.0001) of microglial processes, and the microglial phenotype switched from a normal ramified surveillance state to an activated hypertrophic state (Fig. 7B–F). Then, the mice were treated with the miR-155-5p antagomir or agomir. The results revealed that the miR-155-5p antagomir could abrogate the increased number of Iba1-immunoreactive cells (*p* = 0.0163) and increase the total (*p* < 0.0001) and mean length (*p* < 0.0001) of microglial processes. However, the effect of the miR-155-5p agomir was completely opposite. Moreover, there was no significant change among the NTG, NTG + anta NC and NTG + ago NC groups.

**Fig. 8:**
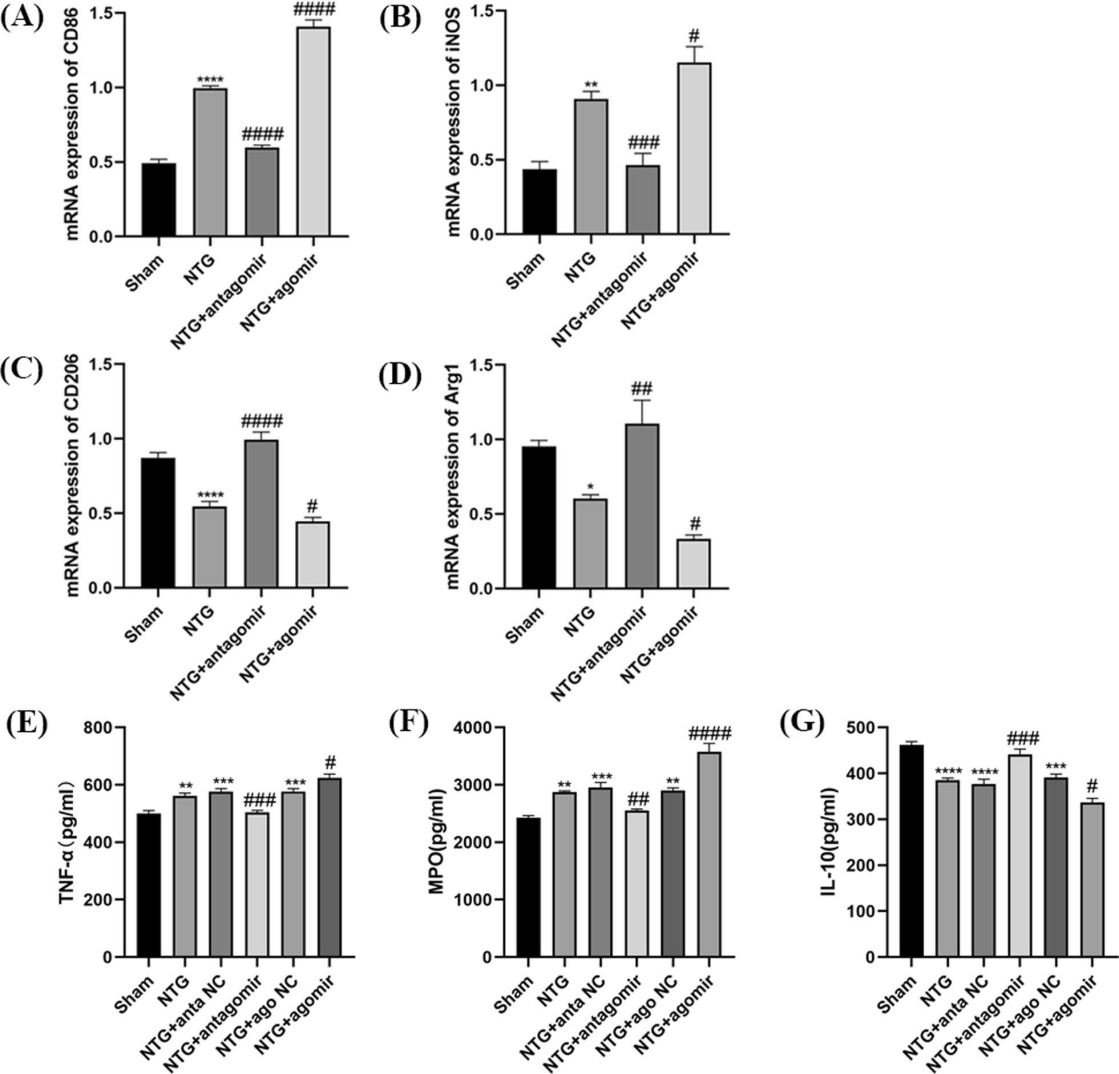
The effects of miR-155-5p antagomir and agomir on microglial polarization and inflammatory substances. **A**, **B**, **C**, **D** The mRNA expression of M1-associated markers (CD86, iNOS) and M2-associated markers (CD206, Arg1). Compared with the Sham group, the mRNA levels of CD86 and iNOS notably increased, while the mRNA levels of CD206 and Arg1 decreased in NTG group. Compared with the NTG group, the mRNA expression of CD86 and iNOS distinctly decreased, while the CD206 and Arg1 levels increased in NTG + miR-155-5p antagomir group. The expression of CD86 and iNOS significantly was higher, while the CD206 and Arg1 levels were lower in the NTG + miR-155-5p agomir group than that in NTG group. **E**, **G**, **F** Compared with the Sham group, the expression of TNF-α,MPO increased, while the expression of IL-10 decreased in NTG group. Compared with the NTG + anta NC group, the expression of TNF-α and MPO decreased, while the IL-10 level increased in miR-155-5p antagomir group. The expression of TNF-α and MPO were higher in NTG + miR-155-5p agomir group than in the NTG + ago NC group, while the IL-10 level was lower. There were no significant differences among the NTG, NTG + anta NC and NTG + ago NC groups. (n = 5–8 in each group; *p < 0.05, **p < 0.01, ***p < 0.001 and ****p < 0.0001 compared with the Sham group; #*p* < 0.05, ##*p* < 0.01, ###*p* < 0.001 and ####*p* < 0.0001 compared with the NTG + NC groups)

Mounting evidence certificated that the M1 microglial phenotype leads to aggravation of the inflammatory response and exacerbation of injury, whereas the activation of M2-like microglia dampens inflammation and promotes repair processes [41, 42]. qRT-PCR was used to assess the balance between the M1 and M2 phenotypes of microglia. We observed that the mRNA levels of typical M1-associated markers (CD86, iNOS) were increased in the NTG group, while the mRNA levels of M2-associated markers (CD206, Arg1) were substantially decreased in Fig. 8A–D. However, miR-155-5p antagomir inhibited the upregulation of CD86 (*p* < 0.0001) and iNOS (*p* = 0.0008) and the downregulation of CD206 (*p* < 0.0001) and Arg1 (*p* = 0.0025) induced by repeated NTG injections, suggesting a switch from the inflammatory M1 phenotype to the anti-inflammatory M2 phenotype in microglia. In addition, the mRNA levels of CD86 (*p* < 0.0001) and iNOS (*p* = 0.0446) were significantly higher and CD206 (*p* = 0.0417) and Arg1 (*p* = 0.0304) were lower in the NTG + miR-155-5p agomir group than in the NTG + ago NC group (Fig. 8A–D).

The changes in inflammation-related indices (TNF-α, MPO, IL-10) were measured using ELISA kits. After recurrent injections of NTG, the protein levels of TNF-α (*p* = 0.0066) and MPO were increased (*p* = 0.0029) in Fig. 8E and F, while the expression of IL-10 (*p* < 0.0001) was decreased (Fig. 8G). However, these changes were abolished by treatment with the miR-155-5p antagomir, resulting in lower TNF-α (*p* = 0.0006), MPO (*p* = 0.0082) expression levels than those in the NTG + NC group (Fig. 8E, F). After miR-155-5p agomir administration, the expression of TNF-α (*p* = 0.0428) and MPO (*p* < 0.0001) was higher than that in the NTG + ago NC group, and the protein level of IL-10 was slightly lower (*p* = 0.0215). There was no significant change among the NTG, NTG + anta NC and NTG + ago NC groups. These results indicate that miR-155-5p is closely associated with microglial activation in CM.

### Administration of SRT1720 and EX527 affected hyperalgesia and CGRP in different manners

**Fig. 9:**
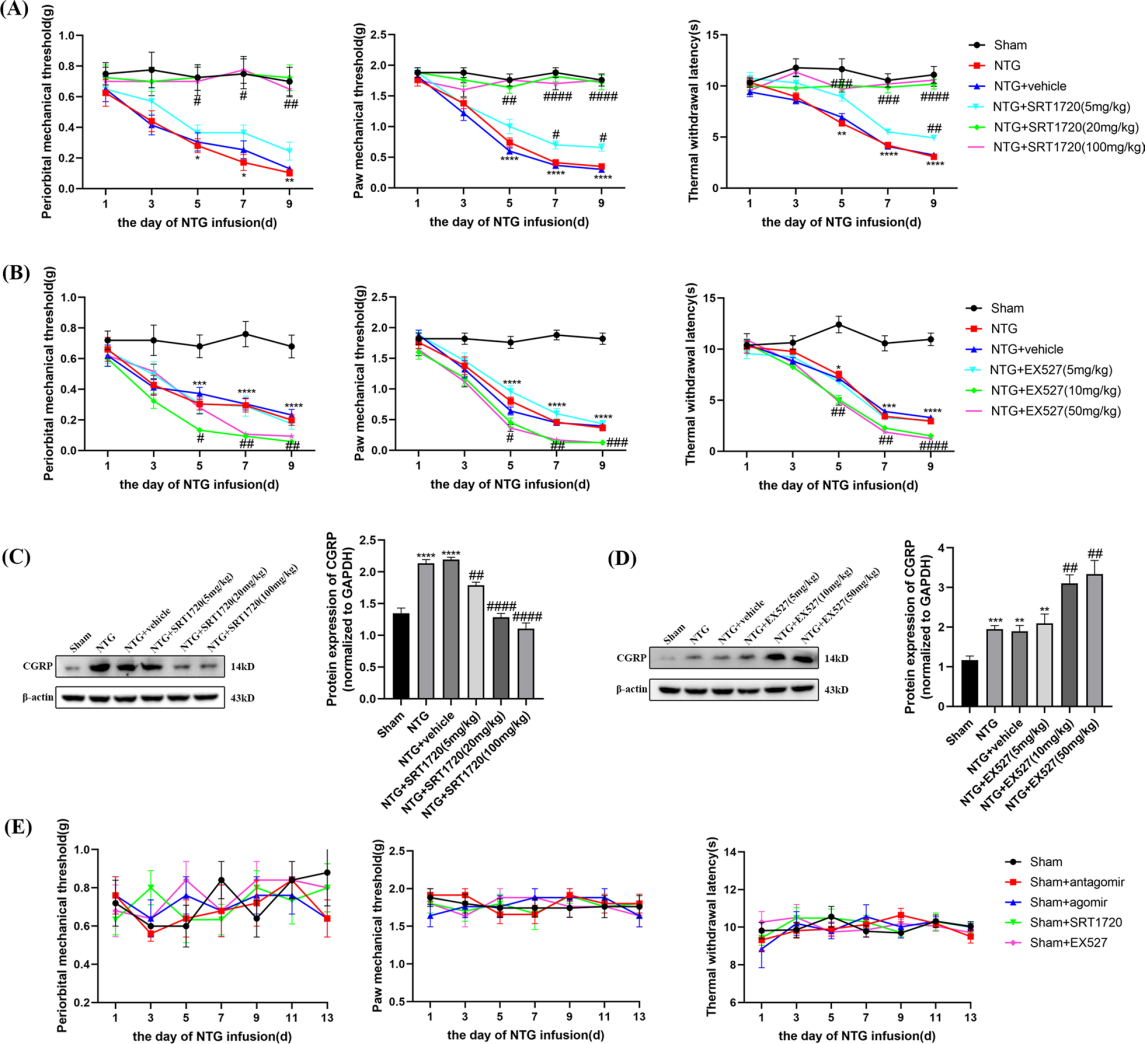
The effects of SRT1720 and EX527 on pain thresholds, and the expression of CGRP. **A**, **B** The mechanical thresholds and thermal withdrawal latency in different groups. **A** Compared with the NTG + vehicle group, 5 mg/kg SRT1720 did not effect, while 20 mg/kg and 100 mg/kg SRT1720 both increased the pain thresholds. **B** Compared with the NTG + vehicle group, 5 mg/kg EX527 did not effect, while 10 mg/kg and 50 mg/kg EX527 both downregulated the pain thresholds. **C**, **D** The protein expression of CGRP in different groups. **C** Compared with the NTG + vehicle group, 5 mg/kg, 20 mg/kg and 100 mg/kg SRT1720 all downregulated the expression of CGRP. **D** Compared with the NTG + vehicle group, 5 mg/kg EX527 did not effect, while 10 mg/kg and 50 mg/kg EX527 both increasing the expression of CGRP. There were no significant differences between the NTG + SRT1720 (20 mg/kg) and NTG + SRT1720 (100 mg/kg) groups, as well as NTG + EX527 (10 mg/kg) and EX527 (50 mg/kg) groups. **E** Mechanical and thermal pain thresholds after miR-155-5p antagomir, agomir, SRT1720, EX527 treatment in the Sham group. No significant difference was observed in the mechanical and thermal pain thresholds among the NTG, NTG + vehicle, NTG + miR-155-5p antagomir, NTG + miR-155-5p agomir, NTG + SRT1720, NTG + EX527 groups. (n = 5–6 in each group; *p < 0.05, **p < 0.01, ***p < 0.001 and ****p < 0.0001 compared with the Sham group; #*p* < 0.05, ##*p* < 0.01, ###*p* < 0.001 and ####*p* < 0.0001 compared with the NTG + NC groups)

To evaluate whether SIRT1 regulates NTG-induced hyperalgesia, SRT1720 or EX527 was intraperitoneally injected every other day for 9 days (five times in total) before the administration of NTG (Fig. 1C). After the administration of a medium (20 mg/kg) or high (100 mg/kg) dose of SRT1720, mechanical and thermal pain thresholds were significantly increased, and the expression of CGRP was decreased compared to those in the NTG + vehicle group, suggesting that the injection of medium and high doses of SRT1720 exerted protective effects (Fig. 9A, C). However, low-dose SRT1720 (5 mg/kg) showed little effect on hyperalgesia or the expression of CGRP. As shown in Fig. 9B and D, there was no significant difference in pain thresholds or CGRP expression after intraperitoneal injection of low-dose EX527 (5 mg/kg) compared with those in the NTG + vehicle group. Exacerbated hyperalgesia and increased expression of CGRP were observed in the middle-dose (10 mg/kg) and high-dose (50 mg/kg) EX527 groups compared with the NTG + vehicle group (Fig. 9B, D). In addition, no significant difference was observed between the middle-dose and high-dose groups of SRT1720 and EX527 groups; thus, the middle-dose was selected for subsequent experiments. Consequently, these results suggest that the upregulation of SIRT1 could relieve hyperalgesia in chronic migraine.

To comprehensively understand the effects of the miR-155-5p antagomir, agomir, SRT1720 and EX527 on mechanical and thermal pain thresholds, eliminating the possible toxic effects of the drugs themselves, healthy mice were individually administered the four drugs. As shown in Fig. 9E, statistical analysis showed no significant differences among the Sham, Sham + antagomir, Sham + agomir, Sham + SRT1720 and Sham + EX527 groups, suggesting that these four drugs have no effect on either withdrawal latency or paw mechanical threshold in the Sham group.

### Treatment with SRT1720 and EX527 influenced central sensitization in the TNC of CM mice

**Fig. 10:**
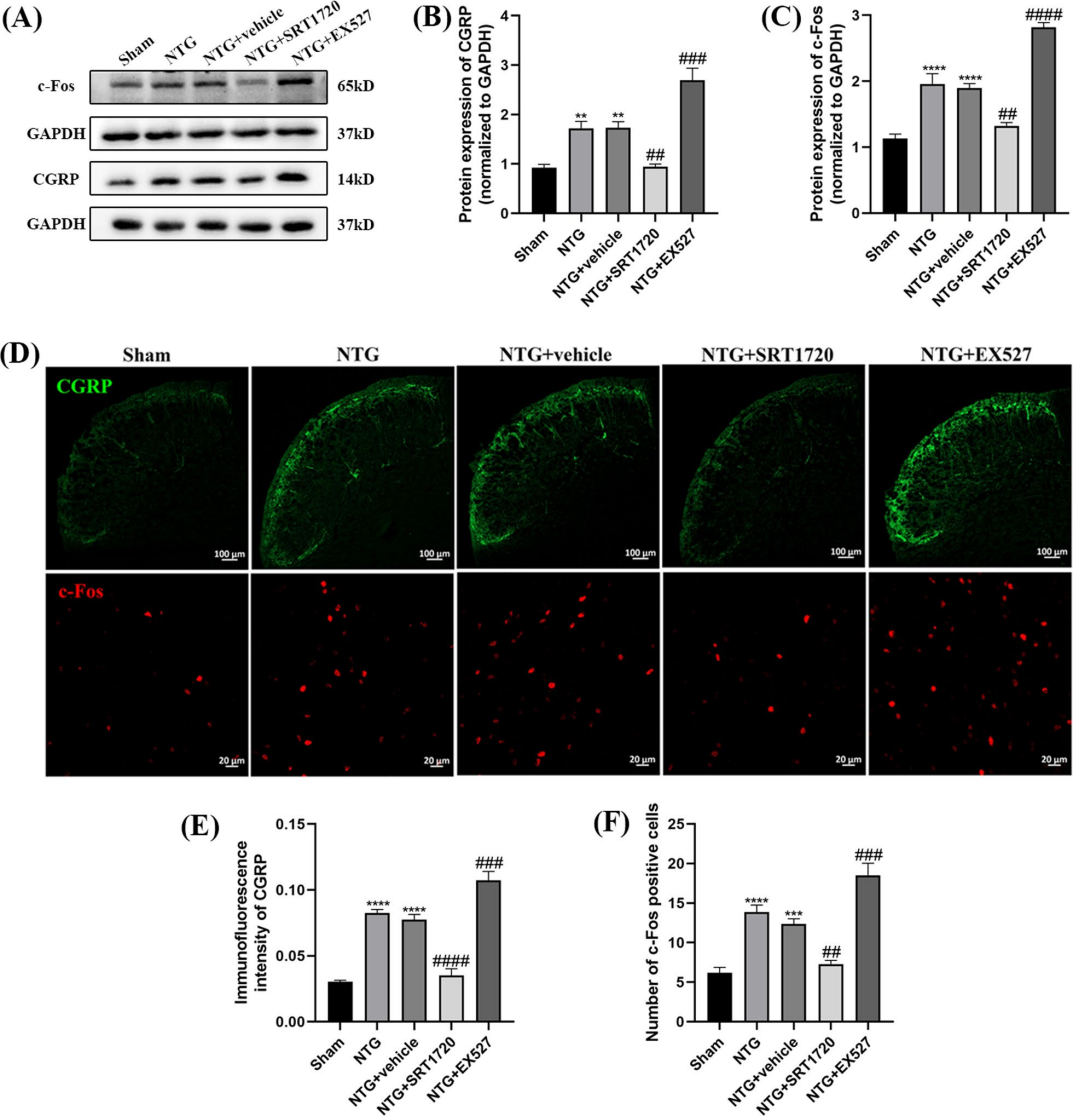
The effects of SRT1720 and EX527 on CGRP and c-Fos expression levels in the TNC. **A**, **B**, **C** Compared with the Sham group, the expression of CGRP and c-Fos increased in NTG group. The CGRP and c-Fos levels in the NTG + SRT1720 group were significantly reduced compared with that in the NTG + vehicle group. The CGRP and c-Fos expression levels were higher in the NTG + EX527 group than that in the NTG + vehicle group. **D**, **E**, **F** The average immunofluorescence intensity of CGRP and the relative number of c-Fos-positive cells both were both higher in the NTG group than in the Sham group. Compared with the NTG + vehicle group, the fluorescence intensity of CGRP and number of c-Fos-positive cells were markedly decreased in the NTG + SRT1720 group. The fluorescence intensity of CGRP and the number of c-Fos-positive cells in the NTG + EX527 group were significantly higher than that in the NTG + vehicle group. There was no significant difference between the NTG and NTG + vehicle groups. (n = 5–8 in each group; scale bar = 20/100 μm; **p < 0.01, ***p < 0.001 and ****p < 0.0001 compared with the Sham group; ##*p* < 0.01, ###*p* < 0.001 and ####*p* < 0.0001 compared with the NTG + NC groups)

To explore whether SIRT1 could regulate central sensitization in NTG-induced chronic migraine, WB and IF were applied to measure CGRP and c-Fos levels in the TNC after the intraperitoneal administration of SRT1720 (20 mg/kg) and EX527 (10 mg/kg). The increased CGRP (*p* = 0.0058) and c-Fos (*p* = 0.0014) were markedly abolished by SRT1720 treatment, while these two indices were higher in the NTG + EX527 group than in the NTG + vehicle group (Fig. 10A–C). The expression levels of CGRP and c-Fos in the IF analysis were consistent with those in the WB results (Fig. 10D–F). No significant difference was observed between the NTG + vehicle and NTG groups.

**Fig. 11:**
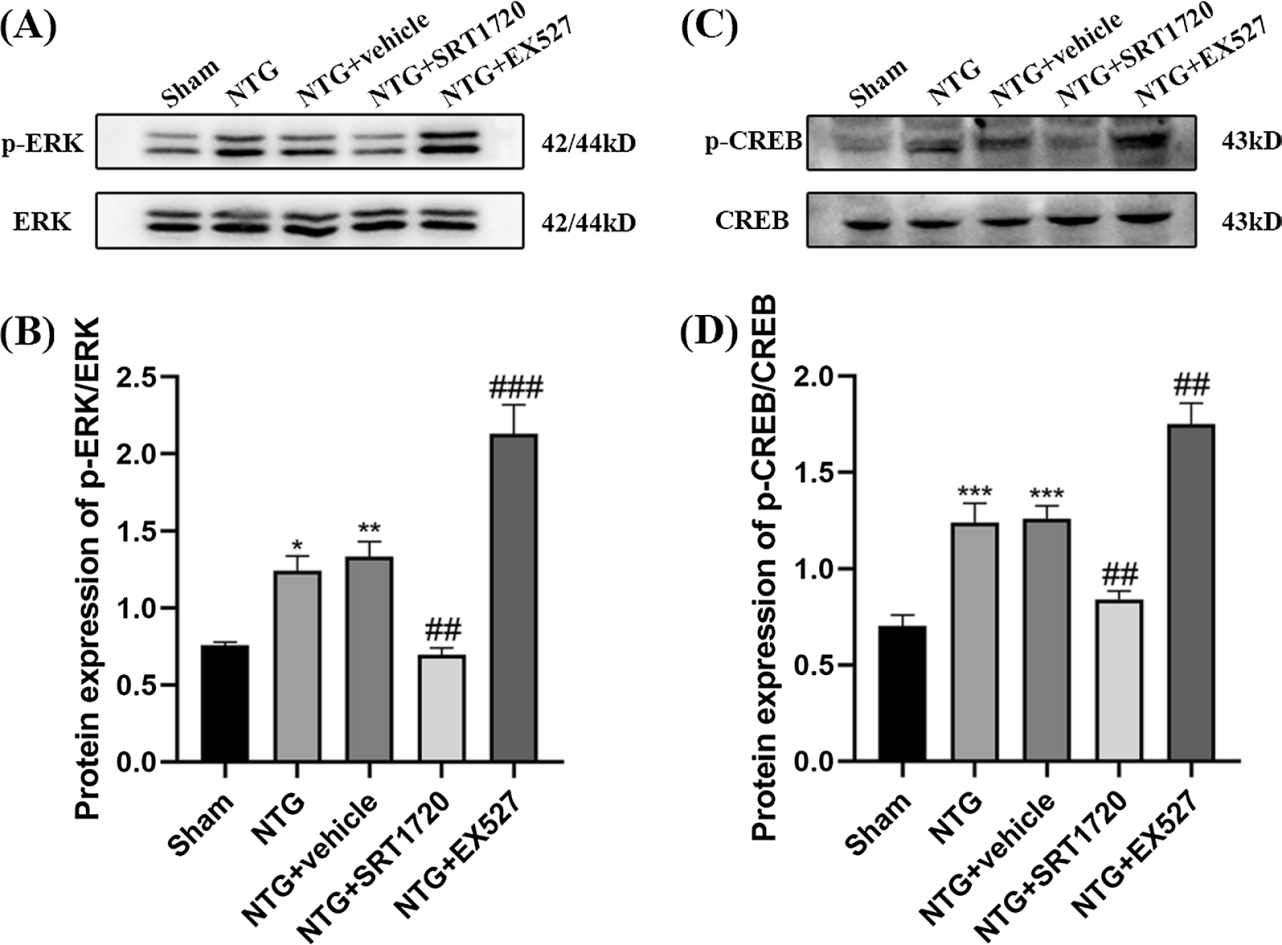
The effects of SRT1720 and EX527 on the phosphorylation of ERK and CREB. **A**, **B**, **C**, **D** The expression of p-ERK and p-CREB-S133 were both higher in the NTG group than in the Sham group. Compared with the NTG + vehicle group, the protein levels of p-ERK and p-CREB-S133 were markedly decreased in the NTG + SRT1720 group. The p-ERK and p-CREB-S133 expression levels were higher in the NTG + EX527 group than in the NTG + vehicle group. The total amount of ERK and CREB remained stable among these groups. There was no significant difference between the NTG and NTG + vehicle groups. (n = 6–8 in each group; *p < 0.05, **p < 0.01 and ***p < 0.001 compared with the Sham group; ##*p* < 0.01 and ###*p* < 0.001 compared with the NTG + NC groups)

The protein levels of ERK, p-ERK, CREB, and p-CREB were measured to assess the activation of neurons. p-ERK (*p* = 0.0277) and p-CREB (*p* = 0.0006) were significantly higher in the NTG group than in the Sham group, and the administration of SRT1720 abrogated the upregulation of p-ERK (*p* = 0.0023) and p-CREB (*p* = 0.0079) (Fig. 11A–D). The opposite changes were found in the NTG + EX527 group. In addition, neither of these drugs caused changes in total ERK and CREB expression. Overall, SIRT1 is associated with central sensitization and activated neurons in NTG-induced CM mice.

### Application of SRT1720 and EX527 was involved in neuroinflammation

**Fig. 12:**
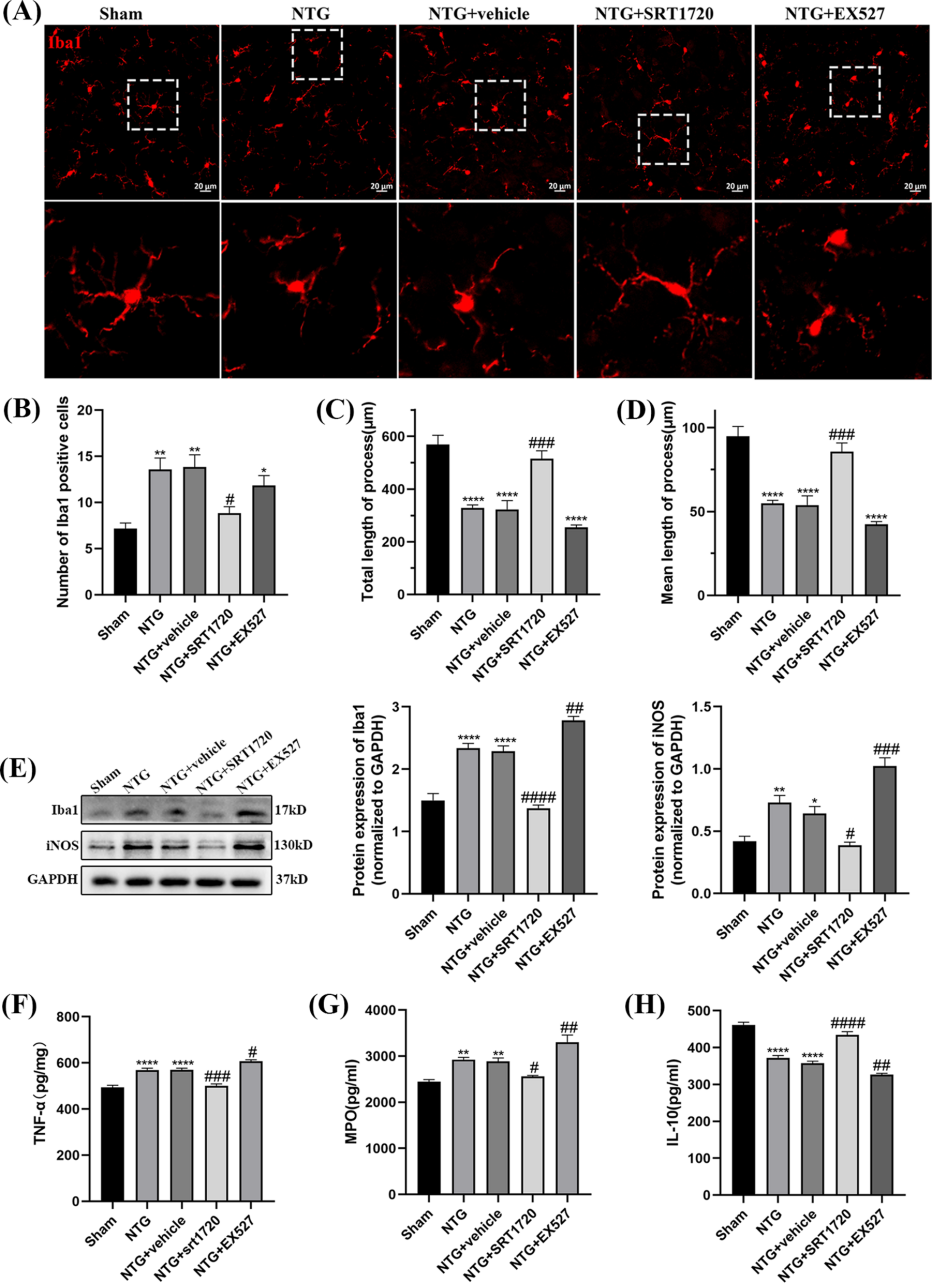
The effects of SRT1720 and EX527 on microglial activation and inflammatory responses. **A**, **B**, **C**, **D** Compared with the Sham group, the number of Iba1-immunoreactive cells increased, while the mean and total length of microglia processes decreased in NTG group. Compared with the NTG + vehicle group, the Iba1-immunoreactive cells markedly decreased, while the total and mean length were higher in NTG + SRT1720 group. There was a little difference in Iba1-immunoreactive cells, total and mean length between the NTG + vehicle and NTG + EX527 groups, without statistically significance. No significant difference was observed between the NTG and NTG + vehicle groups. **E** The expression of Iba1 and iNOS were both higher in the NTG group than in the Sham group. Compared with the NTG + vehicle group, the protein levels of Iba1 and iNOS were markedly decreased in the NTG + SRT1720 group. The Iba1 and iNOS expression levels were higher in the NTG + EX527 group than in the NTG + vehicle group. **F**, **G**, **H** Compared with the Sham group, the expression of TNF-α, MPO notably increased, and the expression of IL-10 decreased in NTG group. Compared with the NTG + vehicle group, the expression levels of TNF-α and MPO were lower, while the IL-10 level was higher in NTG + SRT1720 group. The expression of TNF-α and MPO were higher in NTG + EX527 group than that in the NTG + vehicle group, and the level of IL-10 decreased in NTG + EX527 group. There was no significant difference between the NTG and NTG + vehicle groups. (n = 6–8 in each group; *p < 0.05, **p < 0.01 and ****p < 0.0001 compared with the Sham group; #*p* < 0.05, ##*p* < 0.01, ###*p* < 0.001 and ####*p* < 0.0001 compared with the NTG + NC groups)

To explore whether SIRT1 affects central sensitization by regulating neuroinflammation, the proliferation and morphology of microglia were observed by IF, the protein level of Iba1, iNOS were detected by WB and TNF-α, MPO and IL-10 were measured by ELISA. Increased numbers of Iba1-immunoreactive cells, hypertrophic somatic cells and shortened processes were found in the NTG group (Fig. 12A–D). SRT1720 markedly decreased the number of Iba1-immunoreactive cells (*p* = 0.0206) and increased the total (*p* = 0.0002) and mean length (*p* = 0.0002) of microglial processes, and the phenotype of microglia switched from an activated hypertrophic state to a “resting” ramified surveillance state. Additionally, the total and mean length of microglial processes were slightly shorter in the NTG + EX527 group than in the NTG + vehicle group, but the difference was not statistically significant. In Fig. 12E, the administration of SRT1720 could dismiss the NTG-induced upregulation of Iba1((*p* < 0.0001) and iNOS (*p* = 0.0305). ELISA was used to measure the inflammatory factors TNF-α, MPO, and IL-10 (Fig. 12F–H). Treatment with SRT1720 decreased the levels of TNF-α (*p* = 0.0002) and MPO (*p* = 0.0420) and increased the expression of IL-10 (*p* < 0.0001) compared with those in the NTG + vehicle group, suggesting the beneficial effect of SIRT1 against chronic migraine. Compared with those in the NTG + vehicle group, the protein levels of TNF-α (*p* = 0.0264) and MPO (*p* = 0.0065) were upregulated, and the expression of IL-10 (*p* = 0.0024) was slightly decreased in the NTG + EX527 group. There was no significant difference between the NTG + vehicle group and the NTG group. Taken together, these results suggest that the upregulation of SIRT1 could alleviate neuroinflammation in the TNC of CM mice.

### Administration of SRT1720 alleviated the mir-155-5p agomir-induced inflammatory response and central sensitization.

**Fig. 13:**
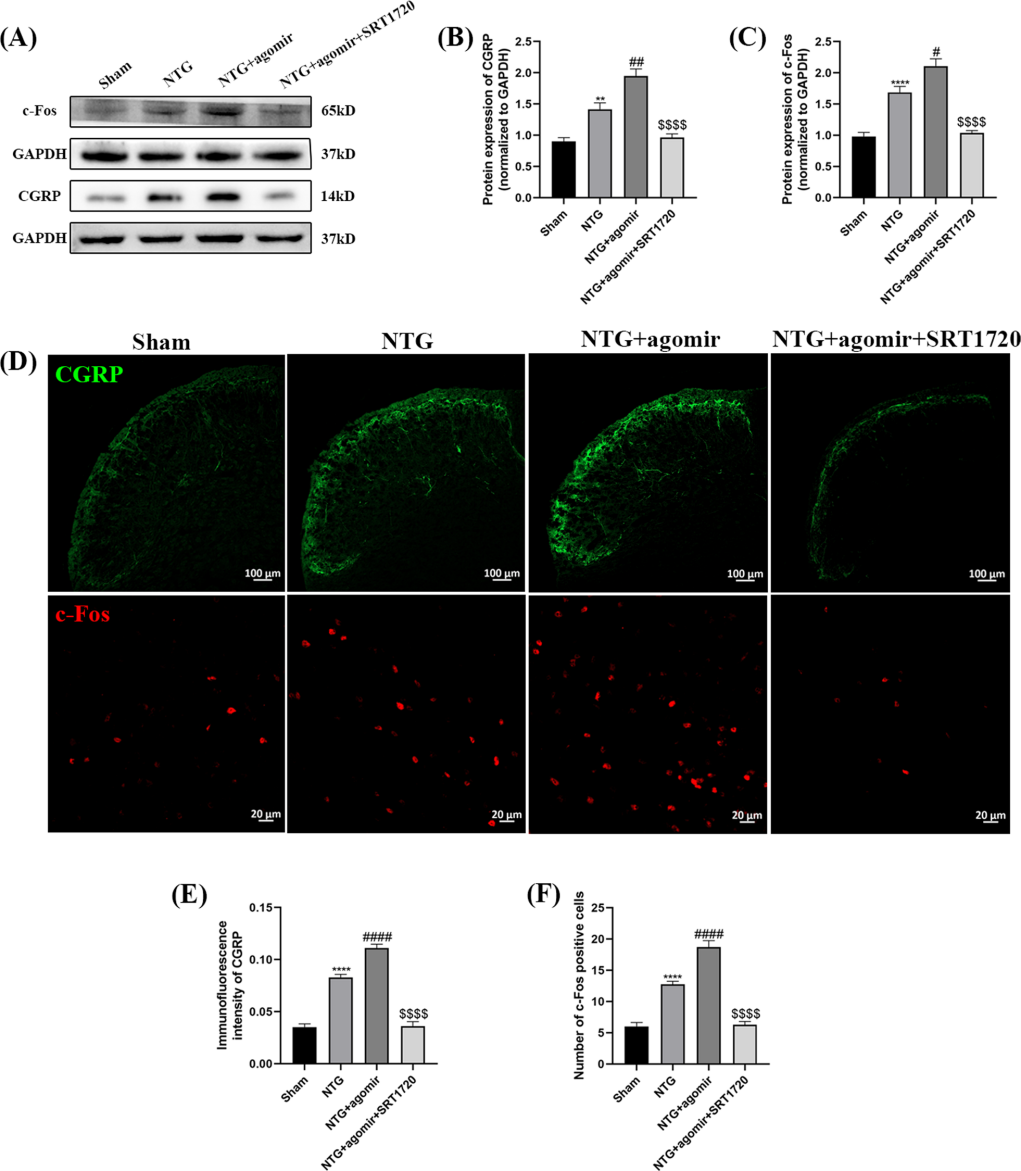
SRT1720 attenuated the miR-155-5p agomir-evoked elevation of CGRP and c-Fos in the TNC. **A**, **B**, **C** The protein levels of CGRP and c-Fos were higher in the NTG + miR-155-5p agomir group than in the NTG group. Compared with the NTG + miR-155-5p agomir group, the expression of CGRP and c-Fos were significantly decreased in the NTG + miR-155-5p agomir + SRT1720 group. **D**, **E**, **F** The immunofluorescence analysis was consistent with WB results. The administration of SRT1720 dismissed the effort of miR-155-5p agomir on the expression of CGRP and c-Fos. (n = 6 in each group; **p < 0.01 and ****p < 0.0001 compared with the Sham group; #*p* < 0.05, ##*p* < 0.01 and ####*p* < 0.0001 compared with the NTG + NC groups; $$$$*p* < 0.0001 compared with the NTG + miR-155-5p agomir group)

**Fig. 14:**
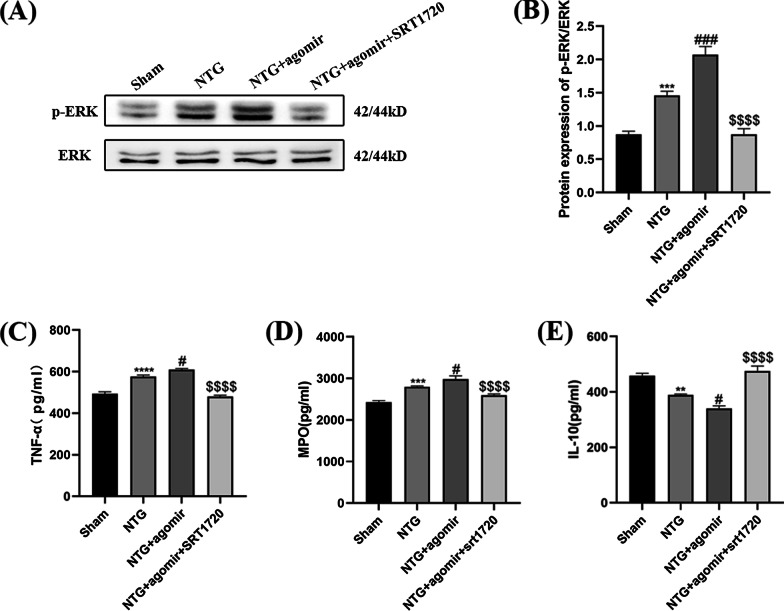
SRT1720 attenuated the miR-155-5p agomir-evoked upregulation of p-ERK, TNF-α, MPO and upregulation of IL-10. **A**, **B** Compared with the NTG group, the p-ERK protein levels were notably increased in the NTG + miR-155-5p agomir group. The p-ERK levels in the NTG + miR-155-5p agomir + SRT1720 group were significantly reduced compared to NTG + miR-155-5p agomir group. **C**, **D** Compared with the NTG group, the TNF-α and MPO levels were slightly increased in the NTG + miR-155-5p agomir group. The TNF-α and MPO levels in the NTG + agomir + SRT1720 group were substantially reduced compared to NTG + miR-155-5p agomir group. **E** The expression of IL-10 in the NTG + miR-155-5p agomir group was lower than that in the NTG group. Compared with the NTG + miR-155-5p agomir group, the IL-10 expression in the NTG + agomir + SRT1720 group was markedly increased. (n = 6 in each group; **p < 0.01, ***p < 0.001 and ****p < 0.0001 compared with the Sham group; #*p* < 0.05 and ###*p* < 0.001 compared with the NTG + NC groups; $$$$*p* < 0.0001 compared with the NTG + miR-155-5p agomir group)

To convincingly verify the key role of SIRT1 in miR-155-5p-regulated inflammation and central sensitization, CM mice were treated with both the miR-155-5p agomir and SRT1720 (Fig. 1D). The expression levels of CGRP, c-Fos, p-ERK and the inflammatory factors were measured. As shown in Fig. 13A–C, compared with the NTG group, the levels of CGRP (*p* = 0.0019) and c-Fos (*p* = 0.0102) increased significantly after the administration of miR-155-5p agomir, respectively. The immunofluorescence results were consistent with the WB results (Fig. 13D–F). However, when the miR-155-5p agomir and SRT1720 were administered together, SRT1720 notably reduced the agomir-induced increase in CGRP (*p* < 0.0001) and c-Fos (*p* < 0.0001) expression. In Fig. 14A and B, SRT1720 also reversed the agomir-induced increase in p-ERK (*p* < 0.0001). The levels of TNF-α (*p* < 0.0001) and MPO (*p* < 0.0001) were lower and the expression of IL-10 (*p* < 0.0001) was higher in the NTG + miR-155-5p agomir + SRT1720 group than in the NTG + miR-155-5p agomir group (Fig. 14C–E). Taken together, these data further suggest that miR-155-5p mediates inflammation and central sensitization through SIRT1.

## Discussion

In our study, we established an NTG-induced chronic migraine mouse model and found increased expression of miR-155-5p and decreased levels of SIRT1 in the TNC. Mechanical and thermal pain thresholds were measured to assess the dependability of our model, and abnormal neuronal activity and central sensitization were confirmed by measuring CGRP, c-Fos, p-ERK, and p-CREB levels. TNF-α, MPO and IL-10 analyses and the immunofluorescence staining images of Iba1 indicated inflammatory responses, and CD86, iNOS, CD206 and Arg1 demonstrated the M1/M2 polarization of microglia. Moreover, treatment with a miR-155-5p antagomir or SRT1720 exerted protective effects against hyperalgesia induced by NTG. However, preventing the activation of SIRT1 or elevating miR-155-5p led to exacerbated symptoms of chronic migraine. Importantly, SRT1720-induced activation of SIRT1 attenuated the exacerbation of neuroinflammation and central sensitization elicited by the miR-155-5p agomir. Our study provides convincing data validating the hypothesis that miR-155-5p promotes neuroinflammation and central sensitization by inhibiting SIRT1 in an NTG-induced mouse model of CM.

NTG, a reliable trigger for migraine, can mimic the clinical characteristics of CM patients [24, 43]. We used a generally accepted chronic migraine mouse model, which was established by repeated intraperitoneal injections of NTG [44]. Migraine is more prevalent in women, and scholars have pointed out that this sex difference may be caused by estrogen [45, 46]. A large number of studies have shown that estrogen fluctuations can regulate transmitters of pain signaling, such as gamma aminobutyric acid and serotonin [47], and mediate neuronal activation [48]. To avoid the interference of estrogen, male mice were selected to establish an NTG-induced chronic migraine model. Given the notable sex differences in migraine prevalence, the sex dimorphism of miR-155-5p involvement in the pathogenesis of CM requires further exploration. Amynah A Pradhan et al. also demonstrated that repeated NTG injections could lead to a progressive decrease in the basal mechanical withdrawal threshold, which could last for one week after NTG cessation [24]. Previous reports have verified that hypersensitivity in the periorbital region and hindpaw could indirectly reflect cephalic and extracephalic cutaneous allodynia [49, 50]. Paw withdrawal latency, paw mechanical threshold and periorbital mechanical threshold were all decreased in our model, which were consistent with the chronic migraine model of repeated dural nociception with inflammatory soup (IS) [51]. Hyperalgesia in the periorbital area innervated by the V1 branch of the trigeminal nerve can represent a specific area of headache response. Although hindpaw allodynia is not a direct indicator of TNC sensitization, it is considered one of the signs of third-order neuron (thalamus) sensitization. Through this pathway, the continuous discharge of TNC neurons inevitably leads to the sensitization of thalamic neurons [12].

Studies in several pain models have shown that miR-155 levels are markedly increased in the spinal cord [18, 52, 53]. Blocking miR-155-5p significantly attenuated thermal hyperalgesia, mechanical allodynia and inflammatory cytokine expression in a neuropathic pain model of chronic constriction injury (CCI) [18]. We confirmed an increase in miR-155-5p expression in the TNC of NTG-induced CM mouse model. The administration of miR-155-5p antagomir reversed the decrease in mechanical and thermal pain thresholds in CM mice, which was consistent with the results of previous studies. These results indicate the vital role of miR-155-5p in nociceptive perception and transmission in the NTG-induced chronic migraine model.

Since the changes in miR-155-5p were consistent with microglial activation in the TNC, we hypothesized that miR-155-5p participates in hyperalgesia associated with chronic migraine by regulating inflammatory responses. Central sensitization may be induced by activated microglia that make neurons produce slow synaptic potentials. Our previous experiments also showed that microglial activation in the TNC area plays a key role in the pathogenesis of CM [11, 14]. Microglial proliferation, hypertrophic cell bodies, and reduced length and complexity could be observed in the TNC of the NTG-induced chronic migraine mouse model. The administration of the miR-155-5p antagomir relieved these inflammatory responses. It has been proposed in the literature that miR-155 could regulate the polarization state of microglia, affecting the progression of neuropathic pain [52]. Our results were consistent with previous studies showing that the mRNA expression of CD86 and iNOS, which are associated with the M1 subtype, increased dramatically and that CD206 and Arg1, which are related to the M2 subtype, decreased after NTG injection. When miR-155-5p was inhibited, microglia tended to switch from the M1 subtype to the M2 subtype. Therefore, miR-155-5p regulated the activation and phenotypic changes of microglia in our model.

In addition, after the CM mouse model was established, the expression of TNF-α and MPO increased distinctly, while the protein level of IL-10 was downregulated, indicating an inflammatory response in the TNC. TNF-α is one of the most well-studied inflammatory cytokines and is mainly derived from microglia, and its activation stimulates the infiltration of neutrophils, whose inflammatory product MPO is an important feature of central nervous system inflammation [10]. IL-10, which is produced by M2 microglia and astrocytes, prevents excessive inflammation [10, 54]. Treatment with the miR-155-5p antagomir relieved pain hypersensitivity, causing the downregulation of TNF-α and MPO and the upregulation of IL-10, suggesting neuroinflammation-related hyperalgesia.

The inflammatory cytokines released by microglia bind to specific receptors on the postsynaptic membrane of nociceptive neurons, activating the second messengers Ca^2+^ and cyclic AMP (cAMP), and then ERK is phosphorylated to regulate the excitability of neurons [10]. The cellular mechanism of the crosstalk between microglia and neurons in the TNC remains to be further studied. Our results showed that phosphorylated ERK and CREB levels increased after continuous NTG stimulation. We also found increased c-Fos levels, indicating abnormally excited neurons. The administration of the miR-155-5p antagomir abrogated the activation of p-ERK and p-CREB, as well as the upregulation of c-Fos, while treatment with the miR-155-5p agomir showed diametrically opposite effects, suggesting that miR-155-5p is involved in NTG-induced neuronal activation.

CGRP facilitates nociceptive transmission and participates in central sensitization by developing and maintaining a hyper-responsive state [37]. The concentration of CGRP in the blood and cerebrospinal fluid of migraine patients is elevated. The nuclear protein c-Fos is rapidly expressed in response to various types of noxious stimuli in neurons [55]. Previous studies have shown that the upregulation of p-CREB triggers the pain response by regulating the expression of CGRP and c-Fos [56, 57]. Some scholars point out that increased c-Fos expression is especially obvious in laminae I and II of the TNC in NTG-induced migraine rats [58] or peripheral nerve injury mice model [59]; thus, we selected this region for c-Fos immunofluorescence analysis. Additionally, the drug effect results showed that treatment with the miR-155-5p antagomir reduced CGRP and c-Fos levels, demonstrating that miR-155-5p inhibition could attenuate central sensitization and prevent mice from suffering hyperalgesia. Conversely, the aggravation of central sensitization occurred when miR-155-5p was activated.

Furthermore, we found that the up- and downregulation of miR-155-5p also altered the expression of SIRT1, which was consistent with the previous literature that miR-155-5p could be used as a target gene to regulate SIRT1 and participate in the treatment of major depressive disorder [22]. Some scholars proposed that SIRT1 is coexpressed in neurons and glial cells [60–62]. In this study, SIRT1 was partly colocalized with microglia, neuron and astrocyte in the TNC. However, after the treatment of SRT1720, SIRT1 revealed higher coexpression level with Iba1, while there is no significant difference in the coexpression of SIRT1 and GFAP. The reasons for this discrepancy may be due to the different experimental animal models and the different experimental tissues. In the spinal cord of SCI mice [30] and the hippocampus of mice with LPS-induced neuroinflammation [63], SIRT1 is coexpressed with microglia, mediating related inflammatory responses and participating in their pathological mechanisms. However, we cannot rule out that SRT1720 and EX527 may affect other cells to decrease NTG-induced hypersensitivity. As such, the data generated with SRT1720 and EX527 need to be interpreted with caution. Moreover, because of the systemic administration of SIRT1 in our current study, it would take a long way for SRT1720 and EX527 to target microglial SIRT1 in the TNC. We cannot rule out the potential effect of other pathways for pain suppression such as the trigeminal ganglion and the thalamus, since SIRT1 is also widely expressed in these areas.

We hypothesized that miR-155-5p provoked microglial activation and central sensitization via downregulating SIRT1, thereby participating in the pathogenesis of chronic migraine. Our experiments also illustrated that SRT1720 and EX527 might regulate NTG-induced microglial activation and the release of TNF-α and MPO, influencing pain thresholds in chronic migraine.

More interestingly, SIRT1 activation abolished the inflammatory response and central sensitization induced by the miR-155-5p agomir, providing compelling data that the upregulation of miR-155-5p promotes neuroinflammation and central sensitization through SIRT1 signaling in CM mice. Furthermore, the rescue experiment results verified that SIRT1 was regulated by miR-155-5p, partly compensating for the lack of a dual-luciferase reporter assay.

Our findings add to those from the chronic migraine study in a number of ways. First, we innovatively proposed that miR-155-5p was involved in the pathological mechanism of CM. Furthermore, we studied miR-155-5p-induced inflammatory responses from multiple angles, including proliferation and morphological changes in microglia and the release of inflammatory substances. Finally, our findings supported miR-155-5p-regulated microglial–neuronal crosstalk in CM. Notably, the microglial–neuronal interaction is only a hypothesis based on the existing results and previous literature, and more detailed research and statistical analysis, including coculture experiments, are demanded in the battle against CM.

## Conclusion

Briefly, existing studies have shown that miR-155-5p is involved in the central sensitization of CM. The downregulation of miR-155-5p alleviated hyperalgesia and central sensitization by inhibiting microglial activation via downregulating SIRT1. Therefore, miR-155-5p may be a potential therapeutic candidate for chronic migraine.

## Data Availability

The data used in this article are available from the corresponding author on reasonable request if necessary.

## Abbreviations

NTG: Nitroglycerin
CM: Chronic migraine
miR-155-5p: MicroRNA-155-5p
SIRT1: Silent information regulator 1
TNC: Trigeminal nucleus caudalis
CGRP: Calcitonin gene-related peptide
Iba1: Ionized calcium-binding adaptor molecule-1
ERK: Extracellular regulated protein kinases
CREB: CAMP-response element binding protein
MPO: Myeloperoxidase
iNOS: Inducible nitric oxide synthase
Arg1: Arginase 1
PBS: Phosphate-buffered saline
PFA: Paraformaldehyde
PVDF: Polyvinylidene difluoride
DAPI: 40,6-Diamidino-2-phenylindole
qRT-PCR: Quantitative real-time polymerase chain reaction
WB: Western blotting
IF: Immunofluorescence staining
NC: Negative control
anta: Antagomir
ago: Agomir
